# Investigating the Role of T-Cell Avidity and Killing Efficacy in Relation to Type 1 Diabetes Prediction

**DOI:** 10.1371/journal.pone.0014796

**Published:** 2011-05-10

**Authors:** Anmar Khadra, Massimo Pietropaolo, Gerald T. Nepom, Arthur Sherman

**Affiliations:** 1 Laboratory of Biological Modeling, National Institute of Diabetes and Digestive and Kidney Diseases, National Institutes of Health, Bethesda, Maryland, United States of America; 2 Laboratory of Immunogenetics, University of Michigan, Ann Arbor, Michigan, United States of America; 3 Benaroya Research Institute at Virginia Mason, Seattle, Washington, United States of America; La Jolla Institute of Allergy and Immunology, United States of America

## Abstract

During the progression of the clinical onset of Type 1 Diabetes (T1D), high-risk individuals exhibit multiple islet autoantibodies and high-avidity T cells which progressively destroy beta cells causing overt T1D. In particular, novel autoantibodies, such as those against IA-2 epitopes (aa1-577), had a predictive rate of 100% in a 10-year follow up (rapid progressors), unlike conventional autoantibodies that required 15 years of follow up for a 74% predictive rate (slow progressors). The discrepancy between these two groups is thought to be associated with T-cell avidity, including CD8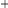 and/or CD4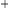 T cells. For this purpose, we build a series of mathematical models incorporating first one clone then multiple clones of islet-specific and pathogenic CD8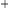 and/or CD4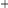 T cells, together with B lymphocytes, to investigate the interaction of T-cell avidity with autoantibodies in predicting disease onset. These models are instrumental in examining several experimental observations associated with T-cell avidity, including the phenomenon of avidity maturation (increased average T-cell avidity over time), based on intra- and cross-clonal competition between T cells in high-risk human subjects. The model shows that the level and persistence of autoantibodies depends not only on the avidity of T cells, but also on the killing efficacy of these cells. Quantification and modeling of autoreactive T-cell avidities can thus determine the level of risk associated with each type of autoantibodies and the timing of T1D disease onset in individuals that have been tested positive for these autoantibodies. Such studies may lead to early diagnosis of the disease in high-risk individuals and thus potentially serve as a means of staging patients for clinical trials of preventive or interventional therapies far before disease onset.

## Introduction

Type 1 Diabetes (T1D) is an autoimmune disorder in which the body's own immune cells (cytotoxic T lymphocytes, CTLs) target the insulin-secreting beta cells in the Islets of Langerhans of the pancreas. These CTLs (including CD8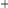 and CD4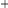 T cells) recognize beta cells and kill them. The process of recognition relies on a complex interaction between a self-molecule peptide, the MHC complex, and T-cell receptors (TCRs). Receptor affinity to peptide-MHC complex (p-MHC) (or T-cell avidity as a whole) varies between different subsets of T cells. High-avidity T cells are implicated in beta-cell destruction, leading to the abolishment of insulin secretion, which is crucial for the regulation of glucose.

The role of T cells as effectors of beta-cell death is undisputed, although formal proof is available mainly in animal models of autoimmune diabetes [1]–[6]. Multiple mechanisms have been invoked to elucidate how beta cells are destroyed. T cells can directly kill beta cells via cell-to-cell contact, through a cytotoxic process, but they can also influence their destruction through other factors, including the release of proinflammatory cytokines, granzyme B, or perforin, and possibly signaling through pathways of programmed cell death [7]–[9]. Several observations suggest that proinflammatory cytokines, such as IL-1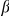, IFN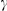 and free radicals are mediators of pancreatic beta-cell death. Autoreactive T cells with potential preferential usage of TCRs responsive to diabetes-related autoantigens may serve as both a potential marker for disease progression and a target for immune manipulation in autoimmune diabetes.

There is also evidence suggesting the involvement of autoreactive regulatory T cells in suppressing islet-specific destructive T-cell activity of differential antigenic specificity locally in the pancreatic draining lymph node, probably via cytokine-mediated modulation of antigen-presenting cells [10]–[12]. In the present work we have elected to model effector T-cell responses because of their close relationship to cytotoxic T-cell-mediated islet injury, proinflammatory cytokine secretion and autoantibody formation.

Although autoreactive CD8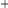 and CD4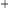 T cells are required for the initiation and progression of the disease, the cellular dynamics leading to disease progression are still not well understood. However, many factors may be combined to determine the risk of T1D disease progression. These include multiple islet autoantibodies, family history of diabetes, genotype (e.g. HLA) and environmental factors. The prognostic significance of any of these risk factors may be modified by the presence or absence of others [13]–[22]. Previous investigative studies have mainly focused on the identification of new immunologic and genetic biomarkers to predict T1D in an effort to facilitate studies in future development of immune-based therapy to treat the disease (see [23], and references therein).

During the progression of the clinical onset of T1D, high-risk individuals exhibit multiple islet autoantibodies and high-avidity T cells. The presence of multiple islet autoantibodies and alleles at the HLA DR and DQ class II loci such as GAD65, IA-2, ZnT8, insulin and cytoplasmic islet cell antibodies (ICA), are considered predictive for the development of clinical T1D among relatives of T1D patients [24]–[30]. The presence of these biomarkers indicates that the autoimmune process leading to pancreatic beta-cell damage has already been initiated. Previous studies have also reported that conventional autoantibody markers (GAD65, IA-2, insulin and ICA), although useful, do not appear to be sufficient in predicting T1D [24], [31]. In fact, recent observations suggest that autoantibodies against the initial 277 amino acid residues of extracellular domain of the neuroendocrine antigen IA-2 in combination with conventional markers can identify rapid progressors of T1D onset when compared to conventional markers alone [23], [24], [32]. This is confirmed in studies showing that in a subgroup of relatives who are positive for GAD65 and novel autoantibodies against the IA-2 epitopes (but not conventional ones) expressed in the extracellular domain of this molecule confers a cumulative risk of 75% at 8 year and 100% by 10 year follow-up. In contrast, the presence of 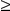
2 conventional autoantibody markers confers a cumulative risk of 58% at 10 years, 63% at 11.5 years and only 74% at 15 year follow-up [24], [27], [31]. The first group was termed rapid progressors and the second group slow progressors.

It has been hypothesized that the pace of the disease in both groups is controlled by the avidity of T cells that react to the same islet-specific autoantigens that autoantibodies react to [30], [33]. In other words, a given epitope on a beta-cell protein will specify the types of autoantibodies and autoreactive T cells that govern the swiftness of the disease. While it is perfectly reasonable to assume such a correlation between autoantibodies and T-cell avidity, the interconnection between these two key factors remains unclear. Here we show that this correlation is also dependent on the efficacy of T cells in killing beta cells, and we examine all possible responses associated with different levels of T-cell avidity and killing efficacy.

Attempts to analyze the interactions between different islet autoantibodies and antigen-specific T-cell proliferation have been hindered by the relatively small numbers of high-risk subjects available for such analyses, as well as the inherent technical challenges in evaluating antigen-specific T-cell proliferation during the pre-clinical stage of T1D. Therefore, we construct here a series of mathematical models to investigate the discrepancy in predicting T1D disease onset exhibited by the two groups (rapid versus slow progressors) based on the notion of T-cell avidity. We initially develop a one-clone model consisting of an antigen-specific population of activated (CD8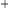 and/or CD4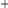) T cells and naïve/mature B cells (plasma cells) to investigate the timing of disease onset. The model identifies regimes in which T-cell avidity and killing efficacy, together with the level of autoantibodies secreted by plasma cells, determine the timing of disease onset. Such studies will be quantitatively helpful in correlating these two notions with the ability of autoantibodies (whose titer levels in blood samples taken from high risk subjects are more easily measured than T-cell avidity and killing efficacy) in predicting the disease. We then extend the model to include two clones of T cells and B/plasma cells each reactive to a different islet autoantigen. The avidity of one of the two clones of T cells is assumed higher, and each clone is divided into low- and high-avidity subclones. The effects of T-cell subclonal competition, within each clone, on disease progression and level of autoantibody are investigated to determine the impact of avidity maturation on beta-cell destruction. The models presented here are related to those in [34]–[39].

## Results

### Mathematical models

#### Full one-clone model

**Figure 1:**
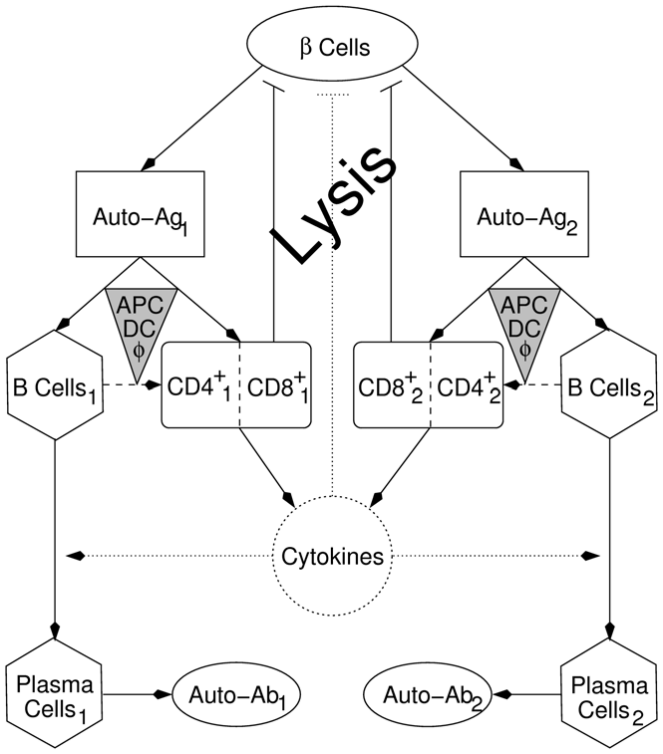
Scheme showing the various factors involved in beta-cell destruction in T1D. Two competing clones of cytotoxic T Lymphocytes, 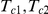 (including either CD8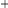, CD4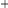, or both), and B cells, 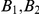, are considered. An initial wave of beta-cell (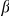) apoptosis and defective clearance trigger autoimmunity by activating several clones of T and B cells via autoantigen presentation (Auto-Ag, 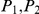) on APCs (gray triangles), including B cells (dashed arrows). Clonal selection and activation of B cells lead to B-cell maturation into immunoglobulin (autoantibody: Auto-Ab, 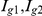) secreting plasma cells, 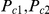, in the presence of various cytokines secreted by effector type CD8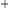 and CD4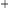 T cells. Beta-cell lysis is amplified by expanding the pool of effector type CD8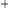 and CD4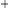 T cells (and their secreted cytokines), forming a positive feedback loop. The dotted circle and arrows indicate that the equation for cytokines is approximated by its steady state, while dashed arrows indicate that direct activation of T cells by B cells is ignored (i.e. the parameters describing T-cell activation by APCs is averaged over the three subpopulations of APCs, including macrophages, DCs and B cells). Eqs. (1a)–(1f) and Eqs. (3a)–(3g) follow this scheme.

Based on the scheme of Fig. 1, we include in this model the following list of cells: insulin-secreting beta cells (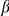); islet-specific autoreactive T cells, including either CD8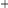, CD4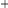 or both (whenever they are reactive to the same autoantigen) (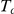); islet specific autoreactive B cells (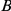); and mature immunoglobulin-secreting B cells or plasma cells (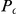). We assume, based on the evidence in [40], that beta cells undergo programmed cell death (or apoptosis) and that defective clearance of dead beta cells by macrophages triggers T- and B-cell activation via antigen presenting cells (APCs). Autoantigens (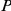) taken up by APCs and expressed as peptide-MHC (p-MHC) complexes expressed on their surface are responsible for the activation of these thymocytes in the lymph nodes of the pancreas. In our previous studies, we have modeled autoantigen processing and p-MHC formation in beta cells and APCs [36], but here we assume for simplicity that such processes are fast and reach steady state rapidly compared to the long time scales studied here (years).

Even though multiple islet-specific autoantigens are implicated in T1D (including insulin, proinsulin, IA-2, GAD, etc.), in this scheme we limit ourselves to only two autoantigens. The activated T cells infiltrate the islets and cause more damage to the surviving beta cells either directly via cell-to-cell contact (by CD8 T cells) or indirectly via harmful cytokines such as IL1-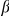 (secreted by CD8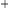 and/or CD4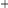 T cells). The destruction of beta cells thus leads to a positive feedback loop that drives the system autocatalytically. We combine the effects of CD8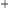 and CD4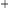 T cells that are reactive to a given autoantigen in one pool and assume, as an approximation, that the secreted cytokines reach steady state rapidly.

On the other hand, immunoglobulin-expressing B cells are also activated by autoantigen uptake (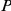) and eventually mature into plasma cells. Plasma cells are efficient producers of immunoglobulin (autoantibodies: 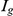) that are released in the circulation. There is a weak evidence for the enrollment of B cells in beta-cell destruction [41], but, as indicated in the scheme of Fig. 1, we ignore such effects. Furthermore, we assume that the vast majority of circulating immunoglobulin is produced by plasma cells with a very small fraction from B cells. B cells may act as APCs in the activation of T cells, but here we assume that the activation is mostly carried out by dendritic cells (DCs) (see below).

By initially focusing on only one clone of T and B cells that are reactive to one given autoantigen, we can, based on the above assumptions, express the scheme of Fig. 1 by a system of six ordinary differential equations, given by
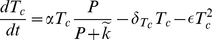
(1a)

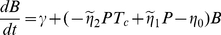
(1b)

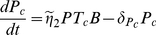
(1c)

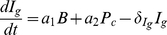
(1d)

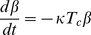
(1e)

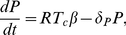
(1f)where 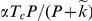 describes the peptide-dependent T-cell self-renewal (which follows Michaelis Menten kinetics), with 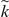 representing the level of peptide for 50% maximum activation, as suggested by [42], [43], 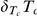 is T-cell turnover, 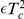 is T-cell homeostasis due to intra-clonal competition (for p-MHC binding sites on APCs and beta cells, since all T cells in this one pool are reactive to the same peptide), 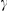 is the basal level of B-cell production from bone marrow, 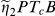 and 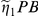 are the peptide-dependent B-cell maturation into plasma cells (via helper T cells whose population size is assumed to be roughly constant, embedded in the value of the parameter 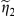 for simplicity) and B-cell self-renewal, respectively (both of which are proportional to the level of autoantigenic peptides expressed on APCs, an interaction required for B-cell maturation and expansion, as elaborated in Assumption 6 below), 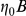 is B-cell turnover, 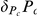 is plasma-cell turnover, 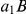 and 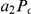 are immunoglobulin secretion from B cells and plasma cells, respectively (presumed proportional to B- and plasma-cell population sizes, with 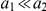 because plasma cells are much more efficient producers of immunoglobulin than B cells),  is immunoglobulin turnover, 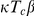 is beta-cell killing by T cells (and harmful cytokines) occurring at a rate 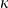 (assumed to be roughly constant during disease progression for a given individual [35], [44]), 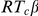 is peptide production (which follows mass-action kinetics [35], [44]) when T cells exert cytotoxicity on beta cells at a rate R per T cell per beta cell, and finally 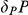 is autoantigenic peptide turnover. The density-dependent intra-clonal competition term 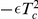 is an essential component of the model, because it guarantees bistability and prevents the the model from having an unbounded expansion in the level of T cells, as we shall demonstrate later.

Implicit in the model are the following assumptions:

1. The T-cell pool is occupied mostly by terminally differentiated effector cells and a small compartment of memory cells responsible for self-renewal. The proliferation rate of this compartment, 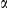, is small and roughly proportional to the population size of APCs, 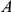, assumed constant (i.e. 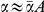). The ability of this memory cell compartment to self-replicate can keep, in certain cases (see below), the level of T cells elevated for prolonged durations extending beyond the time when a critical number of beta cells is lost. We therefore do not include in this model a separate non-vanishing pool of memory T cells to account for a continuously elevated level of T cells (suggested in [45]), an assumption that simplifies the analysis significantly.
2. The inclusion of peptide-dependent thymus input of T cells has been demonstrated to produce similar results to when it is neglected [34]. Therefore, we ignore here the effects of thymus input and focus on self-replication as the only source of newly activated T cells. The observed high level of peripheral autoreactive (high-avidity) T cells in genetically susceptible individuals (carrying, e.g. the INS-VNTR I/I genotype in the case of proinsulin-reactive CD4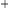 T cells [46]), that escape central tolerance (including the thymus), is accounted for by the initial conditions (i.e. initial level of T cells).
3. T-cell activation is carried out by APCs averaged over three types of cells: macrophages (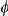), dendritic cells (DCs) and B cells. As suggested by Fig. 1, direct involvement of B-cell pool in activating T cells is ignored in the model. (We demonstrate in the Supplementary Material S1 that discarding this simplifying assumption only moderately alters the general behaviour of the model.)
4. The beta-cell Eqn. (5) is assumed to be a simple decay that depends linearly on the level of effector T cells. We do not include a source term for beta-cell replication or neogenesis, because experimental evidence for such behaviour is lacking. The spatial distribution of beta cells in islets (of Langerhans in the pancreas) is also ignored in this formulation and the total number of beta cells within one pool is considered instead. With such formalism, the stochastic effects of having small number of T cells infiltrating these islets become negligible.
5. As suggested earlier, plasma cells are assumed to be much more efficient than B cells in secreting immunoglobulin (autoantibodies) in the blood circulation (i.e. 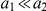). The presence of the very small factor 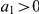 is responsible for the “basal level” of 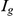 secretion in the absence of plasma cells.
6. Quasi-steady state (QSS) approximation is applied on the cytokines. This makes cytokine concentration (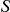) proportional to the population size of T cells (i.e. 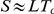). Since B-cell maturation requires both the interaction with APCs (as well as T helper cells, assumed to have constant level), expressing p-MHC complexes on their surface, and T-cell secreted cytokines, this QSS approximation leads to a T-cell- and peptide-dependent B-cell maturation described by the term 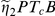.

#### Reduced one-clone model

For simplicity, we focus first on a two-dimensional model of T cells and plasma cells obtained from the full one-clone model above (1a)–(1f). We use the fact that the dynamics of beta cells is very slow on the time scale of T-cell activation, due to homeostatic mechanisms that regulate the beta-cell population [36], [47], i.e. 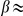 constant (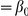). However, as beta cells are killed, autoantigenic peptides rapidly accumulate due to defective clearance, indicating fast peptide dynamics; this justifies a QSS approximation for the peptide ([36], [44] and references therein). For simplicity, we also assume that the rate of B-cell maturation into plasma cells is fast so that QSS approximation can be used on B cells (Eqn. (1b)). Since immunoglobulin has no effect on the dynamics of the model (does not have any pathological effects), Eqn. (1d) can be neglected. In this case, the one-clone model becomes a two-variable model given by



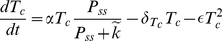
(2a)

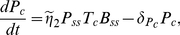
(2b)where 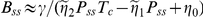, 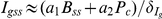 and 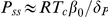.

The two-dimensional reduced model will be used in the next section to understand and illustrate various aspects of the full one-clone model. It possesses a reduced number of parameters (and thus less computational uncertainty) and can be investigated thoroughly using dynamical systems tools that could be helpful in determining how sensitive the model is to parameter perturbations. In particular, we can gain insights by examining the 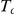
- and 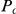-nullclines in the phase plane. We further simplify the analysis by considering scaled versions of the two models derived in Supplementary Material S1. Scaling also reduces further the number of parameters that have to be estimated by identifying parameters that only appear in combination with each other. We use lowercase letters hereafter to denote scaled variables and present our results and simulations in terms of these scaled quantities.

### Coexistence of the healthy and autoimmune states

#### Effects of T-cell avidity on the reduced model

**Figure 2:**
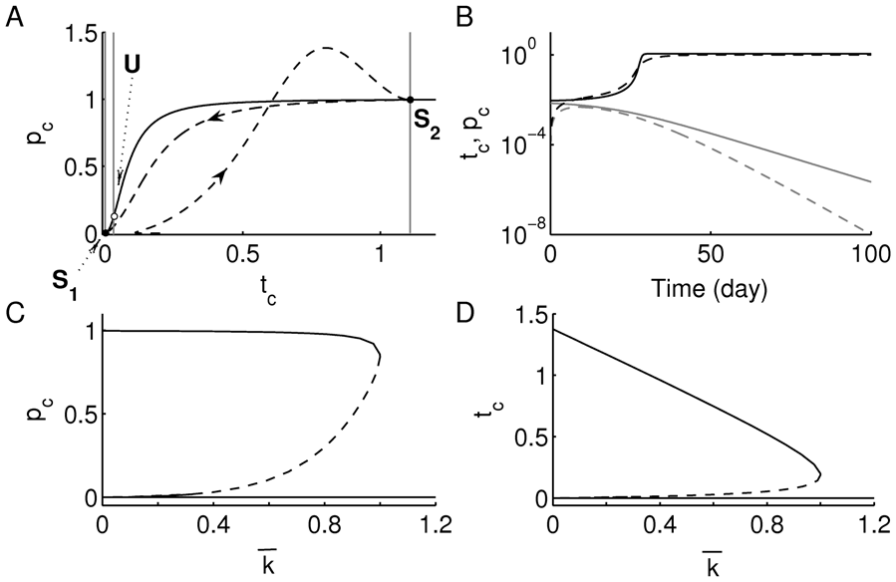
Increasing T-cell avidity (decreasing 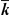) induces bistability. (A) The phase plane of the scaled version of system (2a)–(2b) (see Supplementary Material S1), displaying the 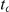
- and 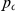-nullclines for 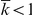 (i.e. for a high level of T-cell avidity). The three vertical gray lines are the 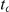-nullclines, while the Hill-like black line is the 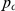-nullcline. Stable steady states 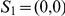 and 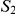 are shown as black dots, while the unstable steady state 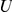 is shown as a white dot. The dashed line traces the time-dependent level of T and plasma cells obtained from the scaled version of full one-clone model (1a)–(1f) superimposed on this phase plane (the arrow-heads represent the direction of flow). (B) The time evolution of 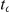 (solid) and 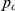 (dashed), approaching the autoimmune (black) and healthy (gray) states, depend on the initial level of T cells (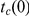). The bifurcation diagrams of (C) 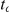, and (D) 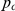 with respect to 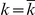 are shown displaying the stable steady states 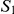 and 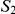 in solid lines and the unstable steady state 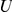 in a dashed line. As demonstrated in Supplementary Material S1, bistability is only observed for 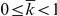, while at 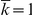, 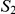 and 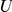 merge together at a saddle node bifurcation point, leaving the healthy state 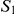 to become a global attractor for 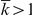 (i.e. for a low level of T-cell avidity).

We follow the ideas of [48] that disease emerges through the existence of a region of bistability. We find as expected (see Supplementary Material S1) that the scaled version of the reduced model (2a)–(2b) exhibits bistable behaviour in which one steady state, 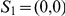, is stable and corresponds to healthy individuals, while the other steady state, 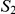, possessing an elevated level of autoreactive T cells (and plasma cells), is also stable but corresponds to type 1 diabetic patients. [In the case of the full one-clone model, 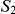 becomes a transient (quasi-stable) steady state, see below.] By considering the points of intersection of the 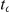
- and 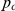-nullclines, we demonstrate in Supplementary Material S1 that these two states coexist whenever 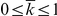, where
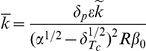
can be considered as the reciprocal of T-cell avidity. Bistability is lost in favor of the healthy state 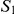 (i.e. 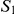 becomes a global attractor) whenever 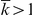. In Fig. 2(A), we show the 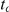
- (gray) and 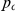
- (black) nullclines of this model. Using the parameter values listed in Table 1, the two states 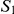 and 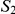 (denoted by the black dots at the intersections of the nullclines) are separated by an unstable saddle point, 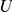 (white dot), whose unstable manifold (the gray vertical line corresponding to the middle 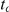-nullcline) is a separatrix between the basins of attraction of the two stable states 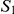 and 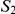 (black dots). Increasing T-cell avidity (decreasing 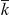) shifts the right-most gray vertical 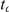-nullcline to the right, thereby increasing the size of the basin of attraction of 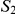 and diminishing the basin of attraction of 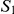, due to a left-shift in 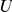 along the 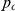-nullcline towards the healthy state. In panel (B), we confirm the bistable behaviour by showing how the time evolution of T cells (solid) and plasma cells (dashed) either rise to elevated levels corresponding to 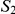 (in black) or decay to undetectable levels corresponding to 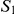 (in gray), depending on the initial values of these two types of cells (which are assumed to have escaped central tolerance). This is illustrated in another way by constructing the bifurcation diagrams of 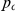 and 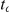 with respect to 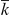, shown in panels (C) and (D), respectively. The dashed line (representing the saddle point) is very close to the lower horizontal solid line (representing the healthy state 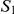) when 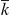 is small. These two panels also show that bistability of 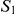 and 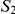 (shown in black lines) is exhibited within the range 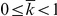, but disappears at 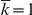 when 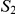 and the saddle point 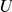 (shown as dashed line) merge together at a saddle-node bifurcation point. Panel (D) shows further that the level of T cells in the autoimmune state increases with increasing avidity, but the level of plasma cells (a read-out for the level of autoantibodies; panel (C)) stays roughly the same.

**Table 1:** Values of the standard parameters appearing in the scaled version (see Supplementary Material S1) of Eqs. (1a)–(1f).

Symbol	Meaning	Value	Range	Ref.
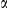	Expansion rate of T cells	4 day 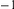	[2]–[20]	[34]–[36],[60]
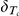	T-cell turnover rate	0.1 day 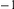	[0.01–0.3]	[34]–[36], [60]
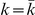	Peptide level for 50%-max activation of T cells	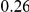	[0–1.4]	[42], [43]
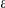	Competition parameter	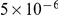 (day  cell) 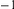	-	[34], [35]
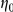	Turnover rate of B cells	0.02 day 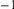	-	[58]
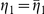	Expansion rate of B cells	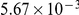 day 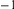	-	[58]
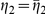	Maturation rate of B cells	2.858 day 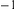	-	[58]
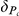	Plasma-cell turnover rate	0.2 day 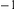	-	[58]
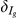	Immunoglobulin turnover rate	0.034 day 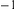	[0.001–0.034]	[58]
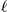	B-to-plasma immunoglobulin secretion ratio	0.1	-	[58]
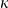	Killing rate of beta cells	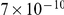 day 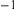	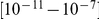	[35], [43]
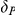	Peptide turnover rate	0.1 day 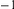		[34]–[36], [44]

Such behaviour has been encountered in previous models involving autoreactive T cells [34]–. The main difference in this model is that the autoimmune state 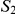 is a transient state that moves as beta-cell number decreases and is tracked by the solution trajectories in the full one- and two-clone models, as we shall demonstrate later.

#### Effects of beta-cell number on the reduced model

**Figure 3:**
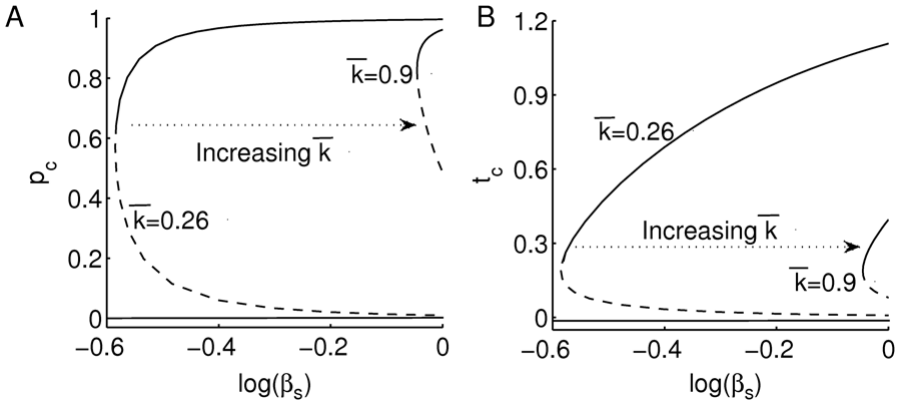
Effects of declining beta-cell number on T- and plasma-cell population sizes. Bifurcation diagrams of (A) 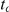 and (B) 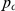 with respect to the parameter 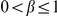 according to the scaled version of system (2a)–(2b) (see Supplementary Material S1) at various levels of T-cell avidity (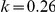 and 0.9), are shown. As in Fig. 2, stable steady states are in solid lines and unstable steady states are in dashed lines. At high T-cell avidity, the range of bistability is large, but the decline in beta-cell number leads to a decrease in T-cell population size.

In the formulation of the (scaled) reduced model used above, the size of the beta-cell population was assumed to be constant as an approximation, because it is slowly varying. However, on longer time scales, the decline in beta-cell number affects T-cell and plasma-cell number because there is less peptide to drive T-cell proliferation (Eqs. (2a)–(2b)). To determine these effects we plot the bifurcation diagrams of 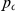 and 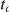 with respect to the parameter 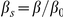, where 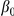 is the initial (normal) number of beta cells. Note that the quantity 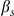 appears in the scaled reduced model as a scaling factor, 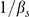, to both 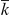, which was used as a bifurcation parameter in Fig. 2, and 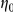 (see Supplementary Material S1). A decrease in 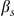, due to the autoimmune attack, causes a left-shift in the right-most vertical 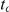-nullcline and a decrease in the steepness of the 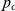-nullcline. The bifurcation diagrams shown in Fig. 3 confirm this outcome when evaluated at two different levels of T-cell avidity: high (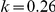) and low (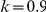). In addition to an increase in the range of bistability between 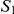 and 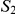 during an increase in T-cell avidity, we observe a decline in the size of the T-cell pool in 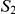 (panel (B)) at high-avidity when 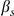 decreases. This behaviour is not exhibited by plasma cells, 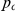 (in panel (A)), whose population size remains roughly the same for a whole range of 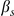 values.

The implications of this behaviour for disease progression and the expression of autoantibodies will become apparent when we study the full one-clone model.

### Dependence of immunoglobulin predictability on T-cell avidity and killing efficacy

#### Model responses and parameter regimes

It has been hypothesized [30], [33] that the discrepancy in the timing of T1D disease onset between rapid progressors and slow progressors, defined as having been tested positive for new and conventional autoantibodies, respectively, is due to the avidity of T cells reactive to the same autoantigenic peptides (epitopes) that autoantibodies react to. In our analysis here, we demonstrate that not only is T-cell avidity a key factor in this process, but also the killing efficacy of T cells, which is a measure of the strength of the apoptotic signal induced inside beta cells by T cells. We assume for simplicity that the killing efficacy is constant for each individual, though it is possible that it varies as the disease progresses.

**Figure 4:**
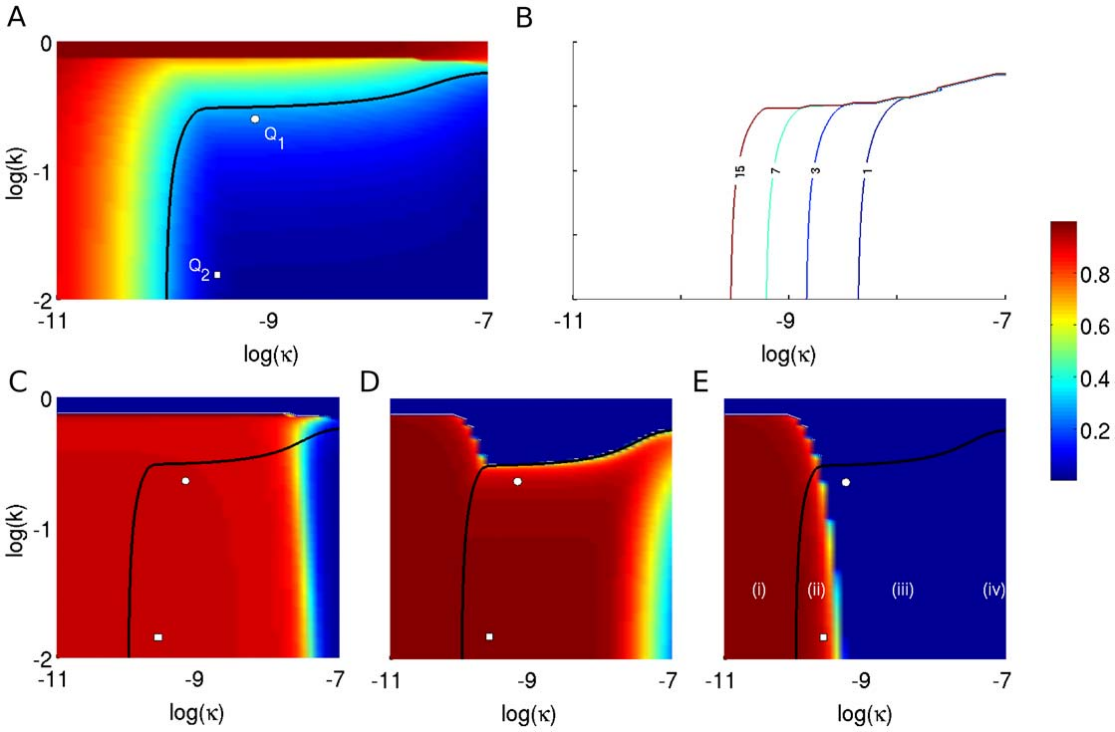
Dependence of T1D disease onset and autoantibody predictability on T-cell avidity and killing efficacy. The simulations here have been generated using the scaled version of the full one-clone model (1a)–(f) (see Supplementary Material S1). Colour represents the level of (A) beta cells, and (C–E) autoantibodies according to the colour-bar on the right. All axes are in logarithmic scale. (A) The steady state level of 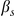 after an autoimmune assault by CD8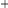 and/or CD4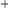 T cells. The black line corresponds to 30% of beta cells remaining (citical threshold). It forms the border line between clinically diagnosed T1D cases from non-diabetic cases. When the level of T-cell avidity is low enough (high 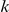 value, dark red region), beta cells remain safe from an autoimmune attack regardless of T-cell killing efficacy. (B) Duration between the emergence of an elevated level of islet-specific autoreactive effector T cells and disease onset (measured in years). Five curves corresponding to 1, 3, 7 and 15 years for disease onset are identified. (C–E) The level of autoantibodies after 6 months of possessing elevated level of T cells (C), at disease onset (D) and at steady state (E) are shown. Four parameter regimes in the 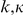-space are identified from these panels (highlighted in panel (E)): A regime corresponding to (i) possessing elevated level of autoantibodies throughout without reaching diagnostic T1D; (ii) possessing elevated level of autoantibodies throughout and reaching diagnostic T1D; (iii) possessing elevated level of autoantibodies until disease onset; and (iv) never possessing elevated level of autoantibodies but still developing T1D. The white circle (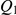) and square (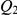) in panels (A), (C–E) correspond to the parameter choices made for 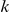 and 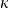 in Figs. 5 and 7.

In order to perform our analysis, we turn our attention to the full one-clone model (1a)–(1f) to view the effects of the autoimmune assault on beta cells. We define the clinical onset of T1D as the time when only 30% of beta-cell number is left (called the critical threshold). While it is true that in most T1D patients, 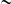
90% of beta cells are lost or become dysfunctional after the autoimmune attack (or at steady state), symptoms of the disease may appear earlier, after 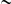
70% of beta-cell loss. (Our analysis remains the same even if the threshold is reduced to a lower value, except for a right and downward shift in the thick black threshold curve shown in Fig. 4(A).) Since thymus input has not been included in this model, we take the initial level of CD8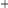 and/or CD4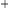 T cells that escaped “central tolerance” to be non-zero.

In Fig. 4(A), we examine the steady-state level of beta cells, using the scaled quantity 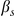, over a range of T-cell avidity (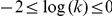) and killing efficacy (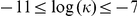). The two quantities 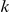 and 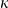, however, are suggested to be inversely correlated with each other for a given T-cell population reactive to a specific autoantigen (i.e. increased avidity would lead to increased killing efficacy) [49]. Since this correlation is quantitatively unknown, we consider here a whole range of values for 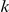 and 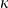 to keep our analysis as general as possible. Our aim is to determine the model response in various parameter regimes and observe the long term effects of simultaneously changing the values of these two parameters on beta cells. We track the steady state-level of beta cells using a spectrum of colours in which red stands for higher levels of beta cells, and blue stands for lower levels, as shown in the calibrated colour bar on the right. The red band at the top part of panel (A) indicates that if the average avidity of CD8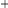 and/or CD4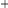 T cells is low enough, i.e. 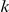 near 1, then beta cells are safe from T-cell destruction regardless of T-cell killing efficacy. Similarly, the left red band on the same panel demonstrates that most beta cells also survive if the killing efficacy is very small, i.e. 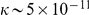, independently of T-cell avidity. However, increasing 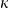 and decreasing 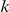 simultaneously, pushes the size of beta-cell population below the critical threshold highlighted by the thick black line separating those that show clinical symptoms of T1D from those that do not. The worst case scenario is shown in the bottom right corner of panel (A), where less than 10% of beta cells survive a strong autoimmune assault by a highly avid T-cell population possessing high killing efficacy. The time to disease onset (i.e. when the 30% threshold is reached) is plotted in panel (B) over the same 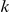-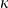 parameter space; as 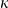 increases, the time to onset decreases from 15 years (slow) to less than 1 year (fast). In other words, the two parameters 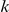 and 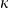 dictate how fast the disease may manifest itself for each individual. (The Supplementary Material S2 shows a movie displaying the gradual loss of beta cells over the same 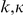-parameter space and over a 30-year period as an illustration.)

We now correlate the landscape of beta-cell survival with the level of circulating autoantibodies at various time points: six months after emergence of a non-zero level of T cells (C), at disease onset for those that become diabetic (D) and at steady state (E). The colour pattern used in these panels is the same as in panel (A). Four distinct parameter regimes (highlighted in panel (E)) can be observed in the 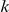-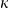 parameter space: (i) The bottom/left corner, left of the critical threshold: The level of autoantibodies remains elevated at all times but without reaching diagnostic T1D (such a scenario may correspond to high-risk subjects who test positive for conventional autoantibodies but never develop T1D); (ii) Middle/bottom regime, right of the critical threshold: As in the previous case, the level of autoantibody here also remains elevated throughout, but individuals in this group eventually develop T1D (this includes both rapid and slow progressors, as shown in panel (B)); (iii) Middle/bottom regime, right of the red region in panel (E): In this case, the level of autoantibodies stays elevated until disease onset, then decays to its basal level (which may not be detectable) at steady state (such a scenario may correspond to rapid progressors who lack elevated level of autoantibodies at later stages of the disease [50]; and (iv) Right/bottom corner, right of the red region in panel (D): Individuals belonging to this regime develop diabetes very rapidly but never become positive for autoantibodies. This peculiar case occurs because the killing efficacy of T cells, 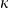, is so large (and may be biologically unreasonable) that a small population of T cells can destroy more than 70% of beta cells. Such behaviour is possible in theory, but 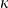 likely cannot be that big.

#### Time evolution

**Figure 5:**
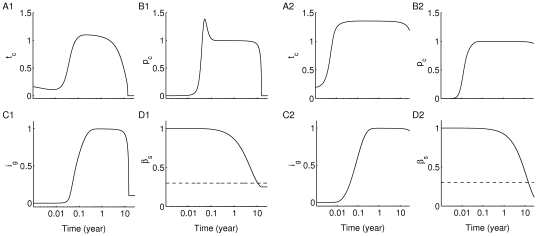
Time evolution of the scaled variables of Eqs. (1a)–(1f). Two parameter combinations (A1–C1) 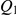: 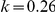, 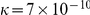 day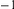; and (A2–C2) 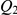: 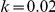, 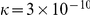 day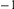, have been used (see Fig. 4). All horizontal axes are in logarithmic scale. The level of (A1–A2) 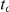, (B1–B2) 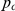, (C1–C2) 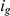 and (D1–D2) 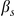 are shown. In the 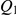 case (A1–C1), the level of islet-specific autoreactive T cells, plasma cells and autoantibody concentration are transiently elevated until disease onset (i.e. when surviving beta-cell level reaches 30% critical threshold), while in the 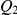 case (A2–C2), they remain elevated for over 60 years (significantly beyond the time it takes to reach the 30% critical threshold). For both parameter combinations, the autoimmune state 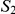 is transiently formed (together with 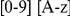) due to an increase in the level of peptide, steering solution trajectories away from the healthy state 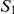 and causing more beta-cell death. When the level of peptide settles down, 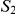 disappears and solution trajectories return back to 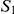 (much faster in the 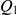 case than in the 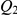 case), generating a loop around the healthy state.

To show typical changes in the level of beta cells and autoantibodies over time for two pairs of parameter combinations for 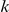 and 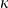, identified by a white circle (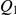: 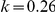 and 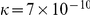 day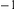) and white square (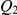: 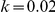 and 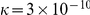 day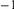) in Fig. 4, panels (A) and (C–E), we simulate the scaled version of Eqs. (1a)–(1f) (see Supplementary Material S1) in Fig. 5. (We call the clone of T cells corresponding to the parameter combination in 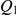 the “standard clone” hereafter.) For the standard clone, the levels of T cells, shown in panel (A1), and plasma cells, shown in panel (B1), initially rise and approach the autoimmune steady state 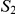, obtained from (the scaled version of) the reduced model (2a)–(2b), due to the fast rise in the level of autoantigenic peptides derived from killed beta cells. When the level of beta cells starts declining, the autocatalytic process driven by T cells also declines (and eventually ceases when beta-cell loss goes below the 30% threshold, shown in panel (D1)), leading to a decrease in the level of T cells and plasma cells. Such a decrease makes 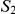 a transient state in the (scaled) full one-clone model. This means that solution trajectories that start from the “basin of attraction of 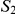
”, would initially and very quickly approach 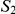 and remain in its vicinity for as long as 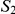 exists (for about 11 years). The decline in the size of the beta-cell population, however, shifts 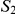 to the left as shown in Figs. 2(A) and 3. If the nullclines in Fig. 2 are plotted as 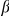 changes (not shown), the solution trajectories of the full scaled one-clone model (dashed line in Fig. 2(A)) remain in quasi-steady state and track along the 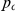-nullcline until 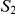 and 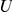 merge together at a saddle-node bifurcation point and disappear (due to a decrease in the level of autoantigen below 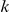, the required amount to keep T-cell replication more dominant over its turnover). At this point, 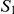 becomes a global attractor. Although the steady state 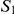 of the reduced one-clone model remains well-defined mathematically as 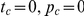, it can no longer be called a healthy state, because it is attained after a massive beta-cell loss.

The level of autoantibodies for this case is shown in Fig. 5(C1), using the scaled quantity 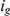, and exhibits similar behaviour as 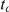 and 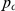. The level of autoantibodies initially rises to an elevated level and stays there for about 11 years before it declines to its basal level. Even though such biphasic behaviour in 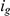 has been observed in NOD mice [50], experimental evidence suggests that the level of autoantibodies remains elevated in most type 1 diabetic human subjects throughout their life span [45]. This behaviour can be obtained from the model using the parameter combination corresponding to 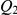 which belongs to parameter group (ii). As shown in the right half of Fig. 5, the levels of T cells 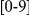 (A2), plasma cells 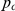 (B2) and autoantibodies 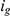 (C2) remain elevated beyond 11 years even though the critical threshold of beta-cell number (shown in panel (D2)) is reached in less than 11 years. If the simulation is continued beyond 60 years, it can be seen that the behaviour of the model for parameter combination 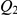 is very similar to that for 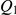. The trajectory again ends up at 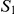, like the dashed line in Fig. 2(A), but it takes much longer to get there. The higher avidity and lower killing efficacy of the T-cell population in the case of 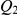, makes it more capable of replicating and destroying beta cells, but it kills more slowly than in the case corresponding to 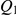, leading to longer T-cell survival for 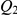 in spite of lower final beta-cell level.

The outcomes described above (which can be validated experimentally) suggest that looking for the high-frequency high-avidity cells may be more successful during the preclinical autoimmune phase of disease, particularly for the more unique specificities such as the IA2 (and maybe ZnT8) targets.

It should be mentioned here that altering the simplifying assumptions 1, 3 and 4 associated with the full one-clone model (i.e. by adding a non-vanishing pool of memory T cells, adding a source term for beta-cell replication/neogenesis, or by making 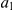 comparable to 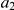), could also result in a maintained elevation in the level of autoantibodies in the four parameter regimes (i)–(iv) defined for Fig. 4.

### Two competing clones

#### Model formulation

**Figure 6:**
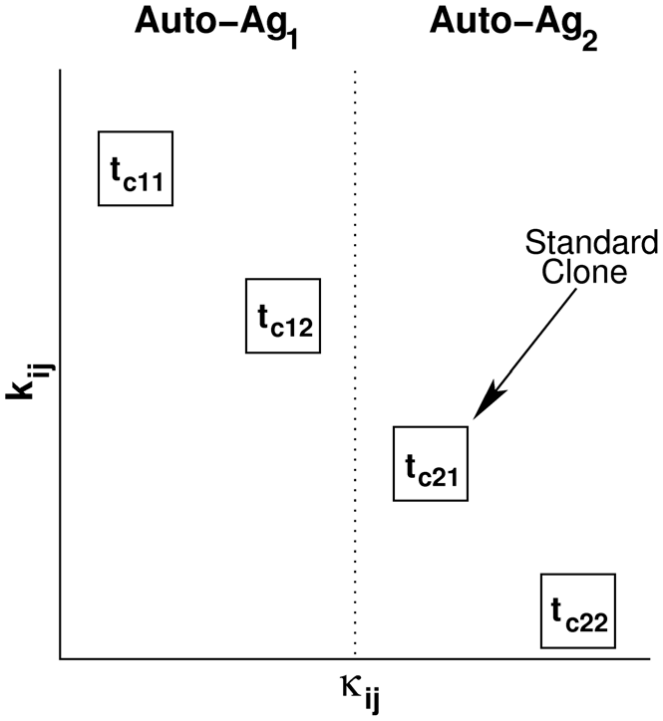
Scheme comparing T-cell avidity, killing efficacy and protein specificity of each subclone under consideration. The vertical and horizontal axes correspond to the reciprocal of T-cell avidity (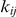) and T-cell killing efficacy (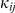), respectively. Auto-Ag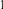 and Auto-Ag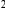 are two islet-specific autoantigens. As shown, the two Auto-Ag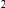-reactive T-cell subclones (in their scaled form), t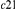 and t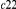, are more avid and more efficacious in killing beta cells than the two Auto-Ag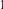-reactive T-cell subclones t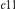 and t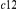 (in their scaled form).

In the two models presented above (the full one-clone and reduced models), we limited autoreactivity to one autoantigenic peptide. In reality, several autoantigenic peptides are involved, leading to multiple clones of T and B cells, each with a given autoantigenic specificity. The interaction of these clones with each other and within themselves (due to the presence of different levels of avidity within each clone) has so far been neglected. We include here a new model which takes into account two clones of T cells that are reactive to two different autoantigens (Auto-Ag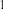 and Auto-Ag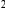), each of which consists of two subclones of high- and low-avidity T cells. Following the scheme in Fig. 1, we obtain the following extended two-clone model
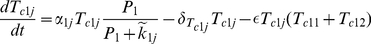
(3a)




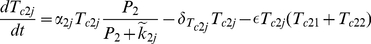
(3b)

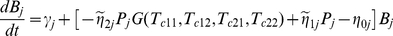
(3c)

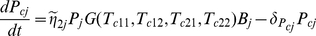
(3d)

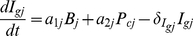
(3e)

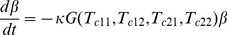
(3f)

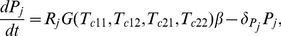
(3g)where 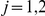 and 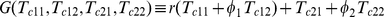. Here 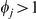, 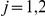, are, respectively, the relative effect of 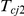 to 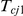 in inducing B-cell maturation and beta-cell killing, whereas 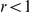 is the relative effect of 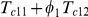 to 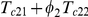 in inducing similar outcomes. In other words, the ascending order of T-cell avidity of these four subclones is assumed to be as follows: 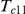, 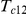, 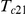, 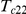 (i.e. 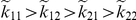), while their ascending order of killing efficacy is: 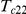, 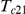, 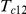, 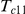 (i.e. 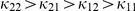), as shown in Fig. 6. (These assumptions are consistent with the inverse correlation thought to exist between 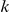 and 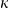, as stated earlier.) The turnover rates of these four subclones, 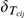, 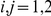, also satisfy: 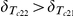 and 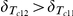 to account for the shorter half-life of the higher avidity subclones and their susceptibility to activation-induced cell death (AICD) (observed in both CD4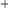 and CD8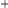 T cells) [49], [51], [52]. Cross competition between T-cell clones with different specificity (i.e. 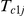 versus 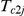, 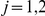), due to limited membrane surface area of beta cells and DCs, is expected to be lower than the direct competition within each clone (whose cells compete for the same p-MHC surface complexes), and therefore would lead to no significant changes in the dynamics of the model if included. Such an outcome has been previously demonstrated in [53] and was confirmed here by numerically testing the effect of cross-competition whose values were lower than 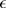. For this reason, we have ignored cross-competition between 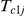 and 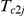 (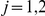) in this model and only considered direct competition within each clone.

#### Model outcomes

**Figure 7:**
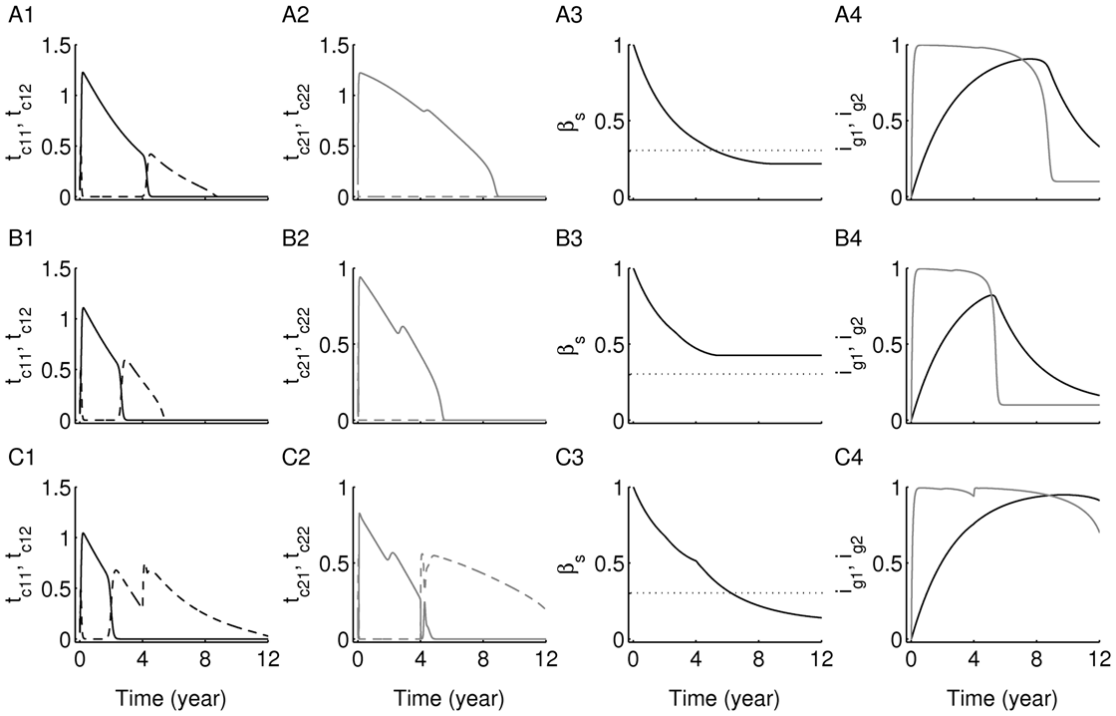
Time evolution of two competing clones of T cells with different antigenic-specificities. The simulations here have been generated using the scaled version of the two-clone model (3a)–(3g) (see Supplementary Material S1). The scaled levels of (A1–C1) the two low-avidity Auto-Ag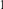-specific subclones: 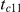 (black/solid) and 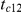 (black/dashed); (A2–C2) the two high-avidity Auto-Ag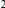-specific subclones: 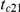 (gray/solid) and 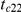 (gray/dashed); (A3–C3) beta cells 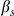 (black solid); and (A4–C4) immunoglobulin specific to: Auto-Ag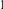, 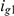 (black), and auto-Ag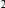, 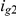 (gray), are shown. Dotted lines in panels (A3–C3) correspond to the 30% critical beta-cell number (threshold) required for preventing clinical symptoms of T1D (staying insulin-independent). Model responses to variations in the value of 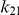 (while keeping 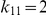, 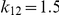 and 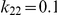 fixed) are simulated to determine the effects of the lower avidity Auto-Ag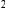-specific subclone, 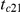, on disease progression. In the upper panels (A1–A4) 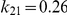; in the middle panels (B1–B4) 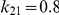; and in the lower panels (C1–C4) 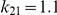. The subclone 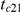 in panel (A2) has the same parameter values as the “standard clone” used in Fig. 5 (white circle). Notice that the levels of autoantibodies, shown in panels (A4–C4), corresponding to the higher avidity Auto-Ag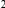-specific subclones (gray lines), become detectable earlier than those corresponding to the lower avidity Auto-Ag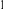-specific subclones (black lines). Also, decreasing the level of avidity of 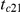 alone in the bottom row (C1–C4), increases the level of beta-cell destruction by reaching steady state level below the 30% threshold (panel (C3)).

In Fig. 7, we investigate the responses of the model defined by Eqs. (9)–(15) to variations in the value of 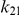 to determine the effects of indirect competition exerted by Auto-Ag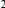-specific subclone, 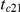 (in gray/solid lines), and its avidity on disease progression. Each row corresponds to a different value of avidity for 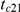 (chosen in such a way that the inequality 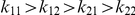 imposed above remains satisfied). The avidities of the Auto-Ag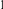-specific subclones, 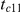 and 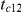 (in black/solid and dashed lines, respectively), and the Auto-Ag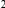-specific subclone, 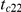 (in gray/dashed lines), are held fixed in each row.

We begin by setting 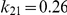, in the upper row (A1–A4). The subclone 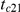 has the same parameter values as the “standard clone” of Fig. 5. Panels (A1) and (A2) show that after a very brief rise in the level of higher avidity subclones within each clone, 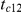 and 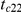 (dashed lines), the lower avidity subclones, 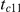 and 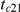 (solid lines), transiently rise, in a manner similar to Fig. 5, to occupy the empty niches left by the disappearing higher avidity subclones and dominate for about 5 and 9 years, respectively. This happens because the 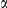
's in the T-cell replication term in Eqs. (3a), (3b) satisfy 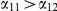 and 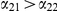, which outweighs the effect of smaller 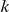 values for an extended period of time. Eventually, however, the level of peptide 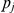 rises above a threshold and the avidity-dependent factor in the replication term dominates, causing the low-avidity sub-clones to decline to undetectable levels. At this point, subclone 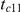 is rapidly replaced by the higher avidity subclone 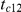, which remains elevated for about 2 years. Such behaviour, consistently observed in all cases discussed below, is consistent with the *switch phenomenon* analyzed in [34]. The combined cytotoxic effects of having three subclones elevated for extended periods of time culminates in a loss of beta-cell number (solid line, panel (A3)) below not only the 30% critical threshold, but also the steady-state level reached in Fig. 2(D1), when the standard clone was considered alone.

Decreasing the avidity of the Auto-Ag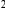-reactive subclone 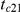 (increasing 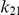 to 0.8) in the middle row (B1–B4) significantly reduces the level of beta-cell destruction shown in panel (B3), an expected outcome in view of the fact that avidity of 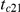 has been reduced (leading to a reduction in the replication of this subclone). The main difference we observe in this case compared to the upper row is that the survival duration of the two subclones 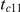 and 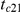, shown in panels (B1) and (B2), respectively, is shorter than that obtained in the previous case (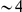 years for the former and 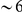 for the latter), unlike the subclone 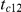 that roughly preserves its survival duration. Even though there is no direct competition between the two main clones of the model, changing the avidity of the Auto-Ag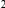-reactive 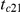 subclone affected T-cell accumulation and survival in the Auto-Ag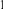-reactive subclones.

The most surprising outcome is obtained in the bottom row (C1–C4), where the avidity of 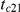 is decreased by increasing 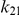 to 1.1. In this case, the combined survival durations of the two clones (first the low-avidity subclone followed by the switch to the high-avidity subclone) is close to 12 years (see panels (C1) and (C2)), leading to more than 70% of beta-cell loss in less than 7 years (shown in panel (C3)). Such an outcome is consistent with what was observed in [49], where low- and high-avidity CD4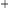 T cells coexisted in T1D patients. In the model, decreasing the avidity of 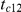 decreases the level of competition with 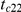 and allows the more damaging subclone to flourish.

Mathematically, the modulation of competition observed as a result of altering 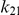 is due to the existence of several transient autoimmune states similar to the state 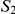 obtained in the reduced one-clone model. Because of the high dimensionality of the two-clone model, these states lie at the intersections of null-hypersurfaces. These change their configurations as 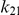 changes, which may lead to an exchange in stability between the autoimmune transient steady states. In the last case, a decrease in competition exerted on 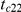 is due to an exchange of stability from a transient state of the form 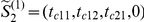 to a transient state of the form 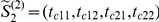.

Notice that in all of these cases, the autoantibodies, shown in panels (A4–A4), that are Auto-Ag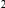-reactive (gray lines), rise to a maximum earlier than the Auto-Ag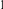-reactive ones (black lines). Such an outcome is expected because it correlates with the avidity and population sizes of the two T-cell clones 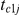 and 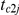, 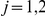, and their corresponding clones of immunoglobulin secreting plasma cells.

**Figure 8:**
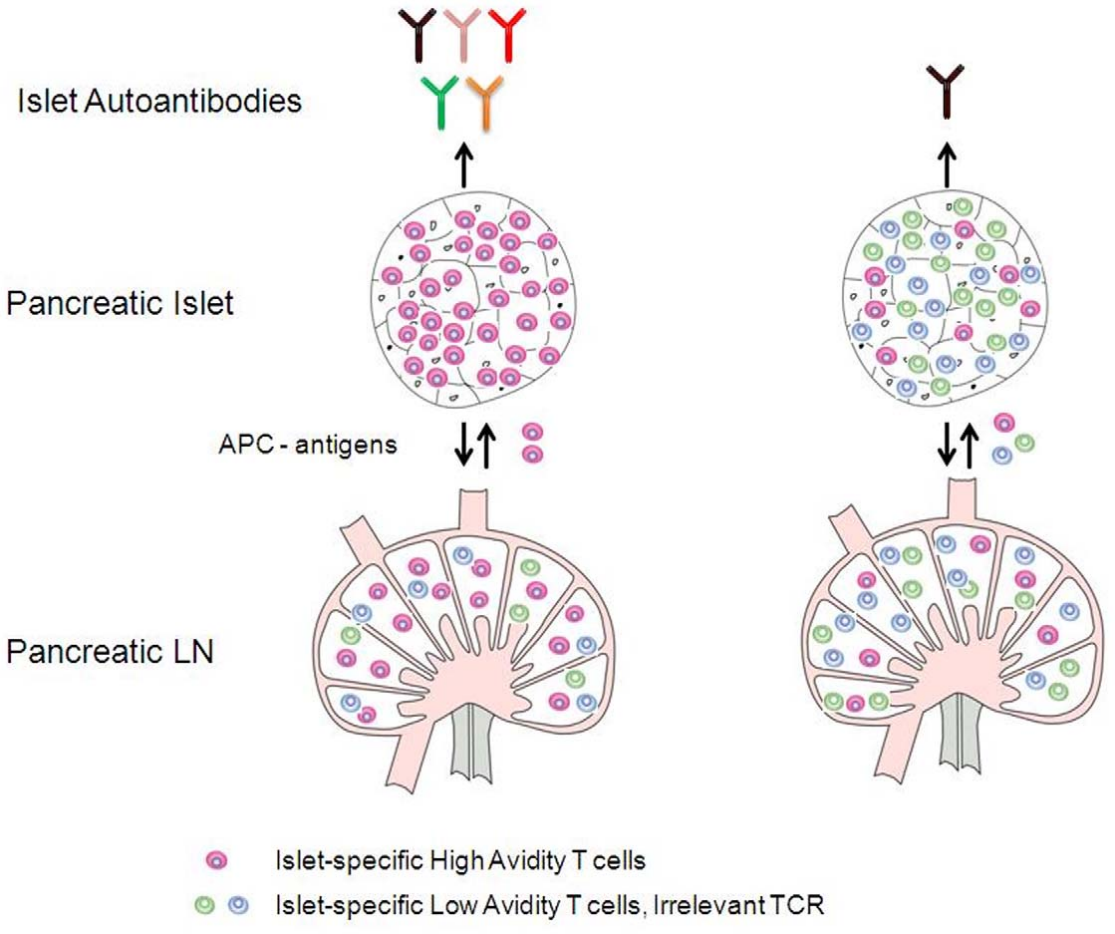
A scheme illustrating the biology of islet-specific T-cell clonal activation and recruitment. The scheme shows T-cell clonal activation in the pancreatic lymph nodes (PLNs), according to their level of avidity, and their recruitment into the islets. The expression of autoantibodies from each step is also shown, where colour diversity represents epitope-specificity of these autoantibodies. Here, the autoantibodies corresponding to the higher avidity T-cell clone are expressed first.

We illustrate these ideas schematically in Fig. 8, showing the activation of low- and high-avidity T cells in the pancreatic lymph nodes (PLNs) via APCs and their recruitment into the islets according to their avidity level. The high-avidity T-cell clones induce their corresponding autoantibodies before other lower avidity clones do. The exclusion of peptide-dependent thymus input from the model may affect the order in which T cells are successively activated and recruited in the islets at decreasing avidities, but the general involvement of these cells and the eventual outcome of the autoimmune attack are independent of such terms.

#### T-cell avidity maturation

The surprising contrast between the first and last cases discussed in Fig. 7 (top and bottom rows) can be reinterpreted using the notion of avidity maturation, a phenomenon observed experimentally in [42] when high-risk human subjects (with one eventually developing T1D) exhibited an increase in the level of avidity of GAD555-reactive CD4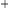 T cells with time. This was demonstrated by observing a left shift (decrease) in the average value of 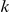 (or EC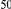) for these cells obtained from blood samples taken at different time points.

We define the quantity
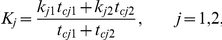
which measures the reciprocal of average avidity over time. A decrease in the value of this quantity corresponds to the avidity maturation observed in the literature.

**Figure 9:**
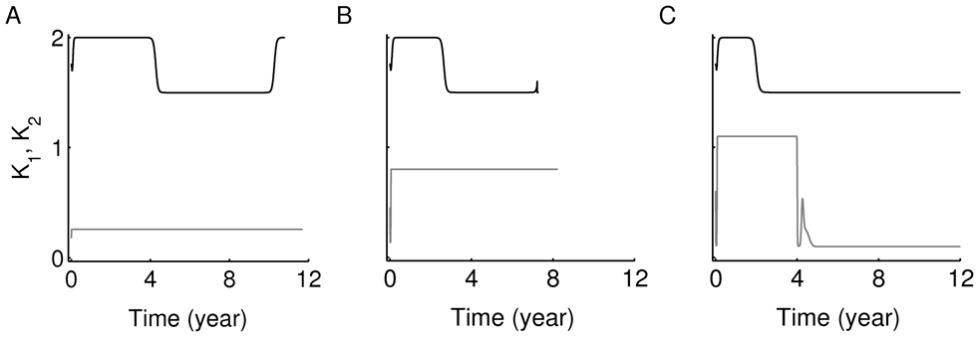
Avidity maturation of the two main clones of T cells considered in Fig. 7. Each clone is reactive to a given autoantigen (either Auto-Ag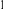 or Auto-Ag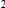, respectively). The average of the reciprocal of avidity, 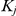, is measured for 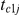 (black) and 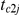 (gray) (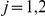) for 12 years using the time evolutions of the four subclones in Fig. 7. Here, panel (A) corresponds to the upper panels (A1–A4) of Fig. 7, (B) corresponds to the middle panels (B1–B4) and (C) corresponds to the bottom panels (C1–C4). The eventual decay of the quantity 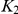, associated with the two subclones 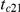 and 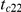, shown in gray in panel (C), to a steady state level lower than the ones reached in panels (A) and (B), indicates increased average avidity relative to (A) and (B), and thus worse impact on beta cells, as demonstrated in panel (C3) of Fig. 7.

We simulate in Fig. 9 the quantity 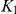 (black) and 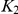 (gray) using the subclones of Fig. 7, where each panel in Fig. 9 corresponds to the similarly labeled row in Fig. 7 ((A) for top, (B) for middle and (C) for bottom row). In both panels (A) and (B), 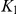 rises rapidly to an elevated level and remains elevated for 3–4 years, then starts declining over time to its steady state level due to the emergence of the subclone 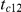, indicating an increase in the average avidity of Auto-Ag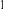-reactive T-cell clone. (The final rises observed at the end of each simulation is inconsequential because they occur when both subclones are at near-zero levels.) On the other hand, the dominance of subclone 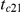, due to its larger 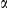-value, in these two cases, causes 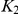 to rise rapidly to its steady state (i.e. 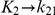) with no avidity maturation. The increase in 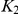 to its steady state level, however, is larger in panel (B) than in panel (A), indicating a population of T cells less effective in destroying beta cells in the former than in the latter. This is consistent with the results in panels (A3) and (B3) of Fig. 7. In panel (C) of Fig. 9, 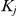, 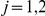, exhibit slightly different behaviour. 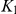 goes to a steady state lower than its initial value after 2 years of transient elevation that is close to that obtained in panels (A) and (B). 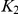, on the other hand, initially rises to an elevated level, then declines to its steady state level in about 4 years, due to a reduced suppression of subclone 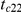 by 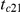. (This competition is mediated by a change in stability as described above.) The steady-state level reached in this case is lower than those attained in Figs. 9(A), (B). Thus, after about 5 years, the Auto-Ag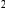-reactive clone has high average avidity (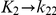) and is also more effective in killing beta cells. This is consistent with panel (C3) of Fig. 7, showing beta-cell number declining below the 30% threshold and exhibiting significantly worse outcomes than those observed in Fig. 9(A). In other words, T-cell competition in the last case led to avidity maturation because of reduction in the avidity of 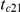 compared to the previous two cases, which increased beta-cell destruction.

## Discussion

During the process of T1D pathogenesis, culminating in beta-cell destruction and overt diabetes, islet autoantibodies manufactured by the immune system and directed against one or more host self-proteins serve as reliable surrogate predictive markers of disease onset. The measurement of islet autoantibodies is now a clear prerequisite in screening for individuals at risk of developing insulin requirement. The presence of two or more of these autoantibodies to islet autoantigens (such as insulin and/or GAD65 or IA-2, or insulin or ICA) can be used as entry criterion for interventional trials [54], [55]. However, the design of these trials should be based on the understanding that about 30% or more of relatives of type 1 diabetics might not develop an insulin requirement within 15 years. Therefore, the prediction of the rate of progression to clinical disease is still difficult. Mathematical modeling may help determine the key risk factors controlling the timing of disease onset in high-risk individuals for early diagnosis and early enrollment in preventative therapies. In this paper, we have taken a first step by investigating two processes, T-cell avidity and T-cell killing efficacy, that underlie both autoantibody levels and the risk of progression. Such modeling can be a useful adjunct to experimental studies because the frequency of autoreactive CD4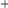 T cells in the peripheral circulation is very low, and the methods for detecting these T cells are laborious, requiring expansion of CD4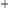 T cells with antigen for 10 days.

We first used a one-clone model to show that not only avidity but also killing efficacy of T cells plays a role in determining the timing of disease onset. These two notions determine the ability of T cells in responding to stimulation (via the level of expression of peptides on APCs) and their ability to induce apoptosis into beta cells. Both of these will have to be taken into account in evaluating the predictive power of autoantibodies reactive to the same epitopes as the T cells. Four different regimes associated with the time course of autoantibody level were identified, depending on the parameter regime specified by the reciprocal of avidity, 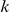, and killing efficacy, 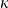 (Fig. 4). In one particular parameter regime (labeled (i), with small 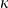 and high-avidity), we found that high-risk subjects may exhibit a high level of autoantibodies from the start of the (weak) autoimmune attack and throughout their whole lives but never develop T1D, a feature consistent with conventional autoantibodies that are hypothesized to be associated with less avid T-cell clones. The three remaining outcomes, on the other hand, were all associated with subjects who eventually developed autoimmune diabetes, but exhibited various behaviours in the time course of their autoantibodies. The first outcome (exhibited by the parameter regime (ii) just right of the critical threshold, with intermediate 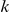 and 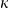 values) expressed elevated level of autoantibodies from the start of the autoimmune attack and remained elevated throughout, a feature consistent with experimental observations associated with novel autoantibodies (such as IA-2 [33]). In the second outcome in regime (iii) (also corresponding to intermediate 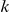 and 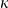 values but with slightly higher 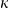), autoantibodies were elevated until disease onset only, then decayed to undetectable level. Such an outcome is similar to what has been observed with Insulin autoantibody (IAA) in NOD mice [50]. Finally, the last outcome in regime (iv) (corresponding to low 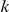 and high 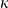 values) exhibited an absence of detectable autoantibodies throughout, which does not commonly occur in high-risk subjects.

The timing of disease onset, defined here as the time it takes to reach the 30% critical threshold, was also evaluated for each of the four cases (regimes) discussed above. The time range for case (ii), which corresponded to high-risk subjects expressing elevated level of autoantibodies throughout, was 7–15 years, depending on the values of 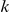 and 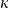, but more so on 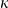. Such a range is consistent with what has been observed experimentally when comparing the predictability of conventional and novel autoantibodies.

We expanded the model to include several competing clones of T cells with different avidities and autoantigenic specificities to shed further light on the role of T-cell intra- and cross-clonal competition on disease progression. The model consisted of two autoreactive T- and B-cell clones specific to two different autoantigens. Each T-cell clone included high- and low-avidity subclones. Using this more complex model, we showed that the maturation of average avidity within each clone determined the level of beta-cell destruction and the level of autoantibodies manufactured by the immune system. For example, in the middle row of Fig. 7, the low-avidity sub-clones came to dominate, resulting in a sub-clinical outcome, whereas in the bottom row, the average avidity of the more avid clone increased (see Fig. 9), resulting in clinical disease. In the latter case, reducing the avidity of the less harmful Auto-Ag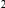-reactive subclone, caused greater loss of beta cells. This was due to a reduction in the competition level within the same clone, leading to an increase in the size of the other competing subclone that was reactive to the same autoantigen. Such complex dynamics is better understood by studying model responses to variations in the avidity ratio within each clone and investigating the impact of such variations in inducing their corresponding autoantibodies. That work will appear in an upcoming manuscript.

In our analysis, we have shown that in most cases, the level of autoantibodies declines to its basal level shortly after the decline of beta cells to levels below the 30% critical threshold needed to prevent the outbreak of clinical symptoms of T1D. The basal level in our model is due to the secretion of immunoglobulin by the inept B cells. As mentioned earlier, this decline in the level of islet-specific autoantibodies is consistent with some of the results obtained from NOD mice in [50]. Experimental evidence in humans, however, suggests that the level of circulating islet-specific autoantibodies remains elevated beyond disease onset, an outcome that is exhibited by our model in a limited parameter regime within the 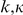-plane. Other possible factors that may account for this type of behaviour have been neglected by our model for simplicity, such as considering a separate pool of memory T cells with long half-life; beta-cell neogenesis/replication; and the possibility of B cells secreting immunoglobulin more efficiently than assumed here, each of which could keep the level of autoantibodies elevated even after the cessation of beta-cell destruction.

Another simplifying assumption in the model is the linear nature of the effect of autoantigen on the transformation of B cells into plasma cells (Eqn. 1b). This is a first degree approximation to Michaelis-Menten type of kinetics like the formulation used for peptide-dependent T-cell activation. This simplifying assumption made the reduced one-clone model easier to analyze. In future efforts, we propose to relax this assumption with the aim of obtaining better correlation between T-cell avidity and autoantibody affinity in predicting disease onset.

There are other possible alternatives for the immunodominance of certain epitope-specific autoantibodies that have not been addressed in the formalism presented here. Examples include: (a) The level of expression of dominant autoantigenic epitopes (such as, GAD65 and aa1-256) on DCs and beta cells could be higher than those less dominant autoantigenic peptides, leading to higher chances of T- and B-cell activation [56]. In other words, epitope dominance may be regulated by the level of expression of autoantigens on DCs and beta cells, rather than by T-cell avidity; (b) ER stress, which may lead to protein misfolding or unfolding in beta cells, could be another factor that determines the dominance of these autoantigenic peptides and their corresponding autoreactive autoantibodies and T cells [36]. That is, such proteins are more inclined to be misfolded/unfolded than others, making them more susceptible to degradation and thus rendering them more immunodominant. Testing such hypotheses will require building models that take into account the various intracellular pathways responsible for such behaviour in beta cells and DCs.

Even though our models presented here (with their limitations) have focused on a subset of factors within a highly complex immunological system, we were successful in testing the hypothesis that there is a correlation between the avidity of islet-specific autoreactive T cells and the risk of developing T1D determined by circulating autoantibodies reactive to the same autoantigenic peptides. That correlation, however, also depends on the killing efficacy of the T cells. Modeling T-cell avidity maturation may shed light on the mechanisms by which benign self-reactive T cells develop into a pathological autoreactive T-cell population during T1D progression, which is potentially of great use given the technical challenges in collecting longitudinal peripheral blood lymphocytes (PBL) to quantify autoreactive T-cell avidities. The models presented here provide a qualitative and quantitative analysis of this correlation and explain the reason for the discrepancy in the timing of disease onset between rapid and slow progressors.

Because many of the parameters of the model are not tightly constrained by experiment, it is important to assess the sensitivity of the model to variation in those values. We have done this in two ways. First, we used the reduced one-clone model, whose repertoire of responses can be fully explored qualitatively by phase-plane analysis. We then expanded this module to build the two-clone model and explored in Fig. 4 the range of behaviors possible under variation of avidity and killing efficacy, two key parameters identified from the phase-plane analysis. A more complete, formal sensitivity analysis [57] of the parameters used in the models presented here, to investigate the impact of various parameters on the general behaviour of the model would be an important future step toward making these models more reliable quantitatively.

Such models may contribute to the development of tools to measure the level of risk associated with each epitope-specific autoantibody and thus prove helpful in diagnosing the disease before irreversible loss of beta cells. That may help us understand the disease process and more accurately identify high-risk individuals at an early stage and enroll them in therapies that can either block the disease or suppress the immune-mediated attack.

## Methods

### Data fitting

To simulate the above models, we must quantify a number of parameters appearing in system (1a)–(1f). Here we show and explain the details of our techniques in estimating these parameters.

In [58], it was stated that the half-life of B cells is 35-42 days and of plasma cells is at least 3–6 days. It follows that 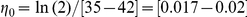 day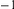 and 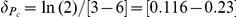 day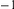. As for immunoglobulin, its half-life varies from one isotype to another [59]. Since IgG is the most dominant isotype associated with T1D, we use its half-life of [7]–[21] day to estimate the parameter 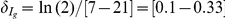 day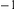.

**Figure 10:**
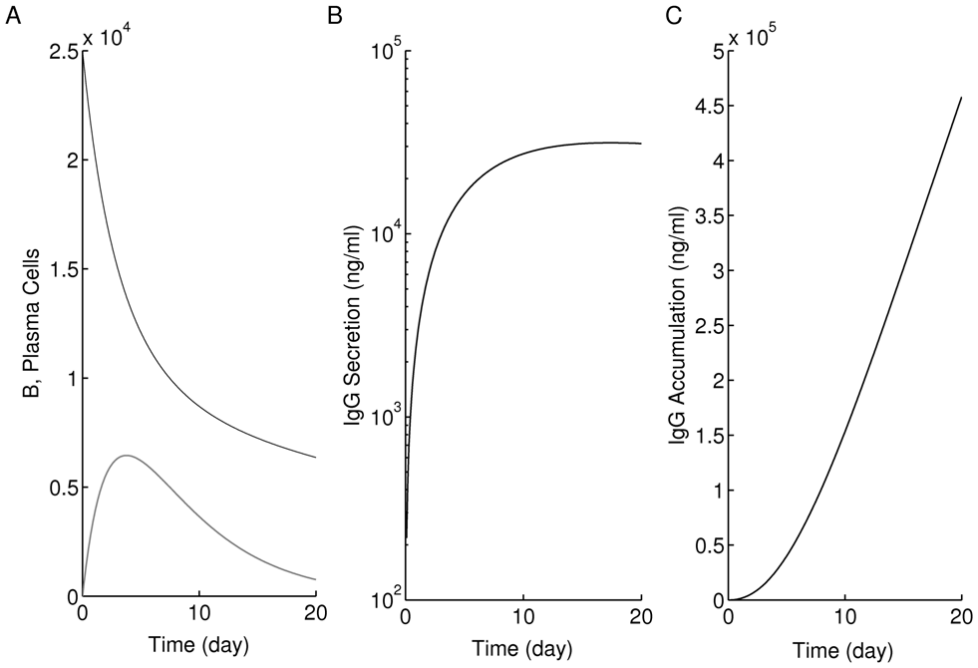
Curve-fitting of B-cell maturation into plasma cells and immunoglobulin (autoantibody) secretion/accumulation. B cells were co-cultured at a density of 25,000 cells/well with 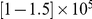 CD3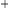 T cells, then IgG secretion rate and accumulation were measured from these cultures [58]. Curve-fitting is applied on (A) the number of B cells remaining (black) and newly formed plasma cells (gray); (B) IgG secretion (vertical axis is in logarithmic scale); and (C) IgG accumulation.

Figs. 1 and 2 in [58] show the time evolution of B-cell maturation into plasma cells and immunoglobulin secretion for 20 days. The data displayed in these figures were collected from co-cultures of B and CD3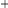 T cells. Based on these cultures, we can design a model that is closely related to the full one-clone model (1a)–(1f), which can be used for data fitting to quantify a few additional parameters. The model in this case is given by
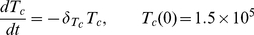
(4a)

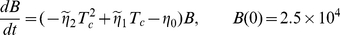
(4b)

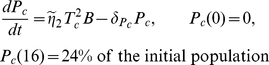
(4c)

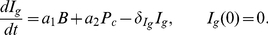
(4d)The simulations of system (4a)–(4d) are displayed in Fig. 10. They were fitted to the curves of plasma cells and IgG secretion/accumulation shown in Figs. 1 and 2 in [58] (the IgG isotype was chosen here because it is the most important for T1D, as stated above). Panel (A) of Fig. 10 shows the level of B cells (black) and mature plasma cells (gray), while panels (B) and (C) show the level of secretion and accumulation of IgG, respectively. The parameter values of Eqs. (4a)–(4d) obtained from this curve fitting are listed in Table 1.

The value of the killing efficacy 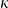 has been estimated to be 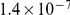 (cell

day)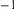 in [35], a value slightly higher than the one that can be deduced using the data in Fig. 5 in [43] (collected ex-vivo). In our analysis, we have considered a wide range for 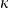 to overcome this uncertainty (see Table 1).

**Figure 11:**
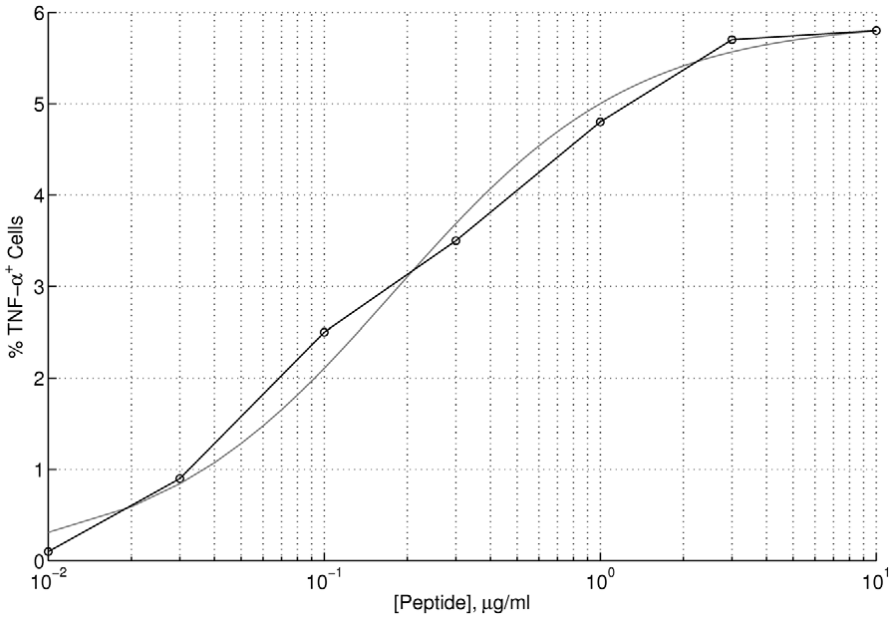
Curve-fitting of T-cell response to autoantigenic stimulation with a given peptide (PPI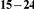). The percentage of CD8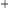 T cells (1E6 clone) staining for intracellular TNF-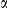 in response to (HLA-A2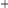) PBMCs pulsed with varying concentrations of peptide was measured in [43] (black line). This dose-dependent behaviour was fitted to a Hill function (gray line) with a Hill coefficient of 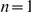. The estimated value obtained for the level of peptide required for 50%-maximum T-cell activation was 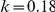 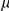
g/ml.

We also know that T-cell activation is controlled by the level of peptide surface expression on APCs (and beta cells). This activation was quantified in both [42], [43]. In Fig. 11, we fitted the T-cell response curve (Fig. 4 in [43]) to increasing level of peptide surface expression to a Hill function, given by 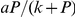, with a Hill coefficient of 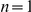. This curve fitting was also done in [42] to estimate the value of 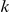 (the level of peptide for 50%-maximum T-cell activation, usually labeled EC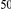). The resulting range for 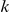 obtained in [42] was 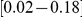 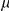
M for high-avidity T cells and 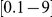 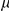
M for low-avidity T cells (with about 95–99% increase in the level of avidity during disease progression in a T1D patient). Our own estimate obtained from the curve fitting in Fig. 11 is 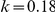 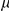
g/ml. A whole range for 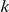 has been considered in our analysis to study the effects of avidity on disease onset.

The remaining parameters of the one-clone model have been previously estimated in [34]–[36], [44], [60] and are displayed in their scaled form (see Supplementary Material S1) in Table 1. As for the parameters of the scaled two-clone model, they were randomly selected from the estimated parameter ranges shown in Table 1, and are listed in Table 2.

**Table 2:** Values of the standard parameters appearing in the scaled version of Eqs. (3a)–(3g) (see Supplementary Material S1).

Symbol	Meaning	Value
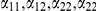	Expansion rates of 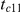 , 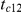 , 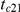 , 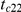 , resp.	7, 6, 4, 2 day 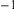
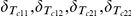	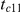 , 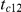 , 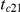 , 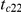 turnover rates, resp.	0.1, 0.15, 0.1, 0.2 day 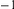
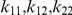 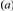	Peptide level for 50%-max activation of 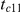 , 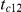 , 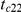 , resp.	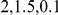
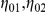	Turnover rates of 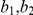 , resp.	0.02, 0.04 day 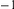
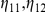	Expansion rates of 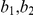 , resp.	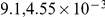 day 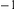
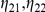	Maturation rate of 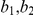 , resp.	7.36, 8.28 day 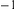
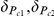	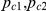 turnover rates, resp.	0.2, 0.4 day 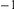
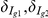	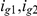 turnover rates, resp.	0.001, 0.034 day 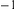
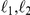	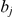 -to- 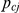 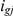 -secretion ratio ( 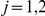 ), resp.	0.1, 0.1
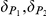	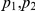 turnover rates, resp.	0.1, 0.1 day 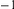
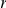	Relative effects of 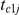 to 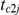 ( 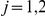 )	0.8
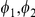	Relative effects of 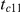 to 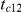 and 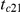 to 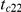 , resp.	1.25, 2

Parameters that are common between the one- and two-clone models are listed in Table 1. 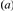 The value of 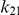 is not listed because it is occasionally altered to observe various outcomes (specified where appropriate).

### Software

The equations of the model were analyzed by phase plane methods, and simulated using MATLAB. The model was also analyzed by linear stability theory and bifurcation methods, as described in Supplementary Material S1. Bifurcation diagrams were plotted using XPPAUTO. We have scaled all models, as shown in Supplementary Material S1, to reduce the number of parameters and to obtain reasonable quantitative results during simulations. Most figures shown here have been generated using the scaled models, unless stated otherwise.

## Supporting Information

Supplementary Material S1 Provides the theoretical analysis used to generate some of the results in the main text.
(0.15 MB PDF)

Supplementary Material S2 The loss of beta cells over 30-year period (for a whole range of T-cell avidity and killing efficacy).
(9.88 MB AVI)
