# Supplementary material for: Investigating the Role of T-Cell Avidity and Killing Efficacy in Relation to Type 1 Diabetes Prediction
Source: PLoS One. 2011 May 10;6(5):e14796. doi: 10.1371/journal.pone.0014796 (PMC3091860; doi:10.1371/journal.pone.0014796)
Supplement: Supplementary Material S1 — Provides the theoretical analysis used to generate some of the results in the main text. (0.15 MB PDF) [file pone.0014796.s001.pdf]

Supplementary Material S1: Investigating the Role of T-Cell Avidity and Killing Efficacy in Relation to Type 1 Diabetes Prediction

Anmar Khadra[1], Massimo Pietropaolo[2], Gerald T. Nepom[3], Arthur Sherman[1]
[1]Laboratory of Biological Modeling
National Institute of Diabetes and Digestive and Kidney Diseases
National Institutes of Health, Bethesda, Maryland, USA
[2]Laboratory of Immunogenetics
University of Michigan, Ann Arbor, Michigan, USA
[3]Benaroya Research Institute at Virginia Mason, Seattle, Washington, USA

A Model Scaling

A.1 Scaled one-clone model

By making the following substitutions: $t_c = T_c/\widetilde{R}$ (here $\widetilde{R} := (\alpha^{1/2} - \delta_{T_c}^{1/2})^2/\epsilon$), $b = \eta_0 B/\gamma$, $p_c = \delta_{P_c} P_c/\gamma$, $i_g = \delta_{I_g}\delta_{P_c} I_g/(a_2\gamma)$, $p = \delta_P P/(R\widetilde{R}\beta_0)$ (here β_0 is the initial number of beta cells), $\beta_s = \beta/\beta_0$, we get

$$\frac{dt_c}{dt} = \alpha t_c \frac{p}{p+k} - \delta_{T_c} t_c - (\alpha^{1/2} - \delta_{T_c}^{1/2})^2 t_c^2 \tag{S1a}$$

$$\frac{db}{dt} = \eta_0 + (-\eta_2 p t_c + \eta_1 p - \eta_0) b \tag{S1b}$$

$$\frac{dp_c}{dt} = \delta_{P_c}\left[\frac{\eta_2 p t_c b}{\eta_0} - p_c\right] \tag{S1c}$$

$$\frac{di_g}{dt} = \delta_{I_g}\left[\ell b + p_c - i_g\right] \tag{S1d}$$

$$\frac{d\beta_s}{dt} = -\kappa \widetilde{R} t_c \beta_s \tag{S1e}$$

$$\frac{dp}{dt} = \delta_P\left[t_c\beta_s - p\right], \tag{S1f}$$

where $k = \delta_P \widetilde{k}/(R\widetilde{R}\beta_0)$, $\eta_2 = \widetilde{\eta}_2 R\widetilde{R}^2\beta_0/\delta_P$, $\eta_1 = \widetilde{\eta}_1 R\widetilde{R}\beta_0/\delta_P$, and $\ell = a_1\delta_{P_c}/(a_2\eta_0)$.

A.2 Reduced/scaled one-clone model

Substituting the variables b, i_g and p by their steady states (fast variables) and assuming that β_s is roughly a constant (slow variable), i.e. $\beta_s = 1$, generates the following two-variable model

$$\frac{dt_c}{dt} = \alpha t_c \frac{t_c}{t_c + \overline{k}} - \delta_{T_c} t_c - (\alpha^{1/2} - \delta_{T_c}^{1/2})^2 t_c^2 \tag{S2a}$$

$$\frac{dp_c}{dt} = \delta_{P_c} \left[\frac{\eta_2 t_c^2}{\eta_2 t_c^2 - \eta_1 t_c + \overline{\eta}_0} - p_c \right], \tag{S2b}$$

where $\overline{k} = k/\beta_s (= k)$ (can be shown analytically to satisfy $0 \leq \overline{k} \leq 1$, see Section B) and $\overline{\eta}_0 = \eta_0/\beta_s (= \eta_0)$.

A.3 Scaled two-clone model

By applying the following substitutions $t_{cj} = T_{cj}/\widetilde{R}$ (here $\widetilde{R} := (\alpha_{21}^{1/2} - \delta_{T_{c21}}^{1/2})^2/\epsilon$), $b_j = \eta_{0j} B_j/\gamma_j$, $p_{c_j} = \delta_{P_{cj}} P_{cj}/\gamma_j$, $i_{gj} = \delta_{I_{gj}} \delta_{P_{cj}} I_{gj}/(a_{2j}\gamma_j)$ and $p_j = \delta_{P_j} P_j/(R_j \widetilde{R} \beta_0)$ $(j = 1, 2)$, we obtain

$$\frac{dt_{c1j}}{dt} = \alpha_{1j} t_{c1j} \frac{p_1}{p_1 + k_{1j}} - \delta_{T_{c1j}} t_{c1j} - (\alpha_{21}^{1/2} - \delta_{T_{c21}}^{1/2})^2 t_{c1j}(t_{c11} + t_{c12}) \tag{S3a}$$

$$\frac{dt_{c2j}}{dt} = \alpha_{2j} t_{c2j} \frac{p_2}{p_2 + k_{2j}} - \delta_{T_{c2j}} t_{c2j} - (\alpha_{21}^{1/2} - \delta_{T_{c21}}^{1/2})^2 t_{c2j}(t_{c21} + t_{c22}) \tag{S3b}$$

$$\frac{db_j}{dt} = \eta_{0j} + \Big[- \eta_{2j} p_j G(t_{c11}, t_{c12}, t_{c21}, t_{c22}) + \eta_{1j} p_j - \eta_{0j} \Big] b_j \tag{S3c}$$

$$\frac{dp_{cj}}{dt} = \delta_{P_{cj}} \left[\frac{\eta_{2j} p_j G(t_{c11}, t_{c12}, t_{c21}, t_{c22}) b_j}{\eta_{0j}} - p_{cj} \right] \tag{S3d}$$

$$\frac{di_{gj}}{dt} = \delta_{I_{gj}} \left[\ell_j b_j + p_{cj} - i_{gj} \right] \tag{S3e}$$

$$\frac{d\beta_s}{dt} = -\kappa \widetilde{R} G(t_{c11}, t_{c12}, t_{c21}, t_{c22}) \beta_s \tag{S3f}$$

$$\frac{dp_j}{dt} = \delta_{P_j} \Big[G(t_{c11}, t_{c12}, t_{c21}, t_{c22}) \beta_s - p_j \Big], \tag{S3g}$$

where $k_j = \delta_{P_j} \widetilde{k}_j/(R_j \widetilde{R} \beta_0)$, $\eta_{2j} = \widetilde{\eta}_{2j} R_j \widetilde{R}^2 \beta_0/\delta_{P_j}$, $\eta_{1j} = \widetilde{\eta}_{1j} R_j \widetilde{R} \beta_{0j}/\delta_{P_j}$, and $\ell_j = a_{1j} \delta_{P_{cj}}/(a_{2j} \eta_{0j})$ (recall that G is linear).

B Theoretical Results

B.1 Nullclines and steady states

We focus in this section on the reduced model described by Eqs. (S2a)-(S2b) to find its steady states and determine under what conditions these steady states are stable. In order to do so, we examine the t_c and p_c-nullclines and their points of intersections (steady states).

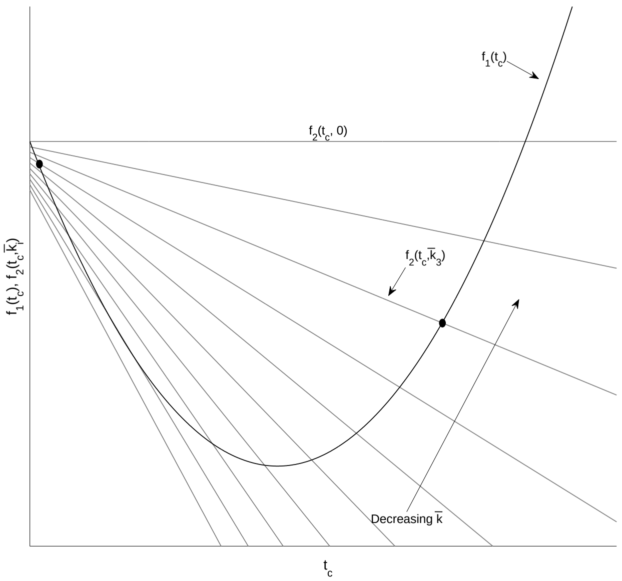

Fig. S1: A sketch of the functions $f_1(t_c)$ and $f_2(t_c, \overline{k})$ for several values of $\overline{k}$. These two functions are guaranteed to intersect at two points for $0 \leq \overline{k} < 1$, but become tangential at $\overline{k} = 1$ and never intersect for $\overline{k} > 1$. The points of intersection are highlighted by black dots for one particular case, where the t_c-component of each point corresponds to the value of the vertical t_c-nullcline, i.e. $t_c = t_{cr}$ defined by Eqn. (S5).

Equation (S2a) is independent of p_c, therefore its nullclines are vertical lines. Clearly, $t_c = 0$ is one t_c-nullcline. For additional t_c-nullclines, we must have

$$\begin{aligned} &\alpha \frac{t_c}{t_c + \overline{k}} = \delta_{T_c} + (\alpha^{1/2} - \delta_{T_c}^{1/2})^2 t_c = 0 \qquad \iff \\ &(\alpha^{1/2} - \delta_{T_c}^{1/2})^2 t_c^2 - (\alpha - \delta_{T_c}) t_c = -\overline{k}\left[\delta_{T_c} + (\alpha^{1/2} - \delta_{T_c}^{1/2})^2 t_c\right]. \end{aligned} \tag{S4}$$

Let $f_1(t_c) := (\alpha^{1/2} - \delta_{T_c}^{1/2})^2 t_c^2 - (\alpha - \delta_{T_c}) t_c$ and $f_2(t_c, \overline{k}) := -\overline{k}\,[\delta_{T_c} + (\alpha - \delta_{T_c}) t_c]$. Fig. S1 shows typically the graphs of these two functions (f_1, f_2) intersecting at two points when the avidity of T cells is high

enough (i.e, when $\overline{k}$ is small enough) and do not intersect otherwise. To determine the parameter range for $\overline{k}$ in which the two curves f_1, f_2 intersect, we solve for the roots of t_c from the quadratic Eqn. (S4). By letting $a := \alpha^{1/2} - \delta_{T_c}^{1/2} > 0$ and $b := \alpha^{1/2} + \delta_{T_c}^{1/2}$, we deduce that the roots of Eqn. (S4) are

$$t_{cr} = \frac{a(b - a\overline{k}) \pm \sqrt{a^2(b - a\overline{k})^2 - 4a^2\overline{k}\delta_{T_c}}}{2a^2}. \tag{S5}$$

To obtain real roots, we require the quantity inside the square root to be non-negative, i.e. $(b - a\overline{k})^2 - 4\overline{k}\delta_{T_c} \geq 0$. It follows that

$$\begin{aligned} &b^2 - 2ab\overline{k} + a^2\overline{k}^2 - 4\overline{k}\delta_{T_c} \geq 0 && \iff \\ &b^2 - 2(\alpha - \delta_{T_c})\overline{k} + a^2\overline{k}^2 - 4\delta_{T_c}\overline{k} \geq 0 && \iff \\ &b^2 - 2\alpha\overline{k} + a^2\overline{k}^2 - 2\delta_{T_c}\overline{k} \geq 0. \end{aligned}$$

But $-2\alpha\overline{k} - 2\delta_{T_c}\overline{k} = -2\overline{k}(\alpha + \delta_{T_c}) = -\overline{k}(a^2 + b^2)$. Hence

$$\begin{aligned} &a^2\overline{k}^2 - (a^2 + b^2)\overline{k} + b^2 \geq 0 \quad \iff \\ &a^2\overline{k}(\overline{k} - 1) - b^2(\overline{k} - 1) \geq 0, \end{aligned}$$

which implies that

$$(a^2\overline{k} - b^2)(\overline{k} - 1) \geq 0. \tag{S6}$$

Inequality (S6) is satisfied either when $\overline{k} \geq (b/a)^2 > 1$ or $0 \leq \overline{k} \leq 1 < (b/a)^2$. If $\overline{k} \geq (b/a)^2$, then one of the $t_{cr} < 0$, a physiologically irrelevant case. However, if $0 \leq \overline{k} \leq 1$, then both $t_{cr} > 0$ and the graphs of the two functions f_1, f_2 intersect at either one point (i.e. they are tangential to each other) when $\overline{k} = 1$, or intersect at two points when $0 \leq \overline{k} < 1$, as demonstrated in Fig. S1. Thus, two physiologically relevant t_c-nullclines (vertical lines) are obtained in the interval $\overline{k} \in [0, 1)$.

By solving for p_c in Eqn. (S2b), we obtain the p_c-nullcline, given by

$$p_c = \frac{\eta_2 t_c^2}{\eta_2 t_c^2 - \eta_1 t_c + \overline{\eta}_0}$$

The points of intersection of the t_c- and p_c-nullclines are the steady states of Eqs. (S2a)-(S2b). There are three such intersections; namely, the point $\mathbf{S}_1 := (0, 0)$, corresponding to a healthy state (with no effector CD8$^+$ T-cell, CD4$^+$ T-cell or plasma-cell accumulation); the point $\mathbf{U}$, whose t_c-component is the left

black dot shown in Fig. S1; and the point $\mathbf{S}_2$, corresponding to an autoimmune state (with elevated level of CD8$^+$ T cells, CD4$^+$ T cells and plasma cells), whose t_c-component is the right black dot in Fig. S1. These steady states can all coexist provided that $\overline{k} \in [0, 1)$. We demonstrate below that $\mathbf{S}_1$ and $\mathbf{S}_2$ are stable, while $\mathbf{U}$ is unstable.

Fig. S1 reveals that increasing T-cell avidity (i.e. decreasing $\overline{k}$ within $[0, 1)$) shifts the right black dot of intersection (and thus the corresponding t_c-nullcline) to the right. This shift is accompanied by an elevation in the level of autoreactive T cells in the autoimmune state $\mathbf{S}_2$. The left black dot of intersection, on the other hand, is shifted to the left against the origin, compressing the basin of attraction of the healthy state $\mathbf{S}_1$. Details of these various configurations are explained in detail in the main text.

Notice that the denominator in the equation of p_c-nullcline could be zero (in which case, the p_c-nullcline will have a vertical asymptote). This may lead to an unbounded increase in the level of T cells in the autoimmune state $\mathbf{S}_2$ while varying $\overline{k}$, a feature considered unrealistic biologically (see Fig. S1(a)). To avoid this situation, we impose the condition $\eta_1^2 < 4\eta_2\overline{\eta}_0$

B.2 Stability analysis

The Jacobian matrix of Eqs. (S2a)-(S2b) is given by

$$J = \begin{pmatrix} \dfrac{2\alpha t_c}{t_c + \overline{k}} - \dfrac{\alpha t_c^2}{(t_c + \overline{k})^2} - \delta_{T_c} - 2(\alpha^{1/2} - \delta_{T_c}^{1/2})^2 t_c & 0 \\ \delta_{P_c}\left[\dfrac{2\eta_2 t_c}{\eta_2 t_c^2 - \eta_1 t_c + \overline{\eta}_0} - \dfrac{\eta_2 t_c^2(2\eta_2 t_c - \eta_1)}{(\eta_2 t_c^2 - \eta_1 t_c + \overline{\eta}_0)^2}\right] & -\delta_{P_c} \end{pmatrix}.$$

The eigenvalues of $J|_{\mathbf{S}_1}$ are $\lambda_1 = -\delta_{T_c}$ and $\lambda_2 = -\delta_{P_c}$ both of which are negative, so the healthy state is always stable. In the presence of the two other steady states, the autoimmune state $\mathbf{S}_2$ is also stable while the steady state $\mathbf{U}$ is unstable. The t_c-nullcline passing through $\mathbf{U}$ is the separatrix between the basins of attraction of the two states $\mathbf{S}_1$ and $\mathbf{S}_2$.

B.3 B-cell-dependent T-cell activation

In one of the model assumptions stated in the main text, we ignored the direct role of B cells in activating T cells and assumed that the three types of APCs under consideration (DCs, macrophages and B cells)

act uniformly on the T-cell population. We also assumed that the population size of APCs is roughly constant. Here we show that having a separate pool of B cells that acts directly on T cells as APCs for activation and cell replication, does not significantly alter the general behaviour of the reduced one-clone model.

To varify this, we modify Eqn. (S2a) to account for B-cell activation of T cells, as follows

$$\frac{dt_c}{dt} = (\alpha_B b + \overline{\alpha}) t_c \frac{t_c}{t_c + \overline{k}} - \delta_{T_c} t_c - (\alpha^{1/2} - \delta_{T_c}^{1/2})^2 t_c^2, \tag{S7}$$

where $\alpha_B b t_c^2/(t_c + \overline{k})$ is the B-cell-dependent T-cell activation occuring at a rate α_B and satisfying $\alpha_B b + \overline{\alpha} \approx \alpha$. (This equation derives from the non-scaled form as done before.) Including such terms in the dynamic equation of t_c generates a cubic-shped t_c-nullcline by joining the two right vertical nullcline asscociated with Eqs. (S2a)-(S2b) (see Fig. S2. Increasing the value of a_B decreases the steepness of this cubic nullcline and alightly alters the location of the steady states $\mathbf{S}_2$ and $\mathbf{U}$, but does not their stability. This suggests that the approximation used in Eqn. (S2a) is justifiable.

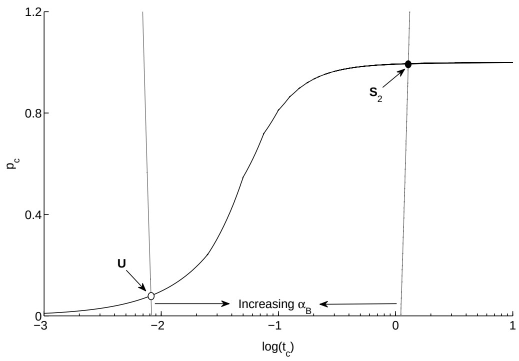

Fig. S2: The phase plane of Eqs. (S7) and (S2b), displaying the t_c- and p_c-nullclines for $a_B = 0.5$ ($t_c = 0$ nullcline is not shown because the $_c$-axis is in logarithmic scale). The two gray lines are the t_c-nullclines, while the Hill-like black line is the p_c-nullcline. The stable steady state $\mathbf{S}_2$, shown as black dot, is the autoimmune state as before, while the unstable steady state $\mathbf{U}$ is shown as a white dot. (The healthy state $\mathbf{S}_1$ is not shown.) Including the term $\alpha_B b t_c^2/(t_c + \overline{k})$ in the dynamic equation of t_c modified the shape of the t_c-nullclines only slightly.
